# A displacement-based finite element formulation for incompressible and nearly-incompressible cardiac mechanics

**DOI:** 10.1016/j.cma.2014.02.009

**Published:** 2014-06-01

**Authors:** Myrianthi Hadjicharalambous, Jack Lee, Nicolas P. Smith, David A. Nordsletten

**Affiliations:** Department of Biomedical Engineering, King’s College London, St Thomas’ Hospital, London SE1 7EH, UK

**Keywords:** Cardiac mechanics, Solid mechanics, Incompressibility, Near incompressibility, Perturbed Lagrangian, Finite element method

## Abstract

The Lagrange Multiplier (LM) and penalty methods are commonly used to enforce incompressibility and compressibility in models of cardiac mechanics. In this paper we show how both formulations may be equivalently thought of as a weakly penalized system derived from the statically condensed Perturbed Lagrangian formulation, which may be directly discretized maintaining the simplicity of penalty formulations with the convergence characteristics of LM techniques. A modified Shamanskii–Newton–Raphson scheme is introduced to enhance the nonlinear convergence of the weakly penalized system and, exploiting its equivalence, modifications are developed for the penalty form. Focusing on accuracy, we proceed to study the convergence behavior of these approaches using different interpolation schemes for both a simple test problem and more complex models of cardiac mechanics. Our results illustrate the well-known influence of locking phenomena on the penalty approach (particularly for lower order schemes) and its effect on accuracy for whole-cycle mechanics. Additionally, we verify that direct discretization of the weakly penalized form produces similar convergence behavior to mixed formulations while avoiding the use of an additional variable. Combining a simple structure which allows the solution of computationally challenging problems with good convergence characteristics, the weakly penalized form provides an accurate and efficient alternative to incompressibility and compressibility in cardiac mechanics.

## Highlights

• A finite element displacement formulation stemming from the Perturbed Lagrangian.
• The efficiency is enhanced with a Shamanskii–Newton–Raphson scheme.
• Comparison with Perturbed Lagrangian, Lagrange Multiplier and penalty methods.
• Presence of locking in the penalty method, for whole-cycle cardiac mechanics.
• The presented form combines good convergence behavior with a simplified structure.

## 1 Introduction

The human heart is a remarkably complex organ, translating cellular ATP consumption into the systemic blood flow [1]. Over the last four decades, computational modeling of cardiac mechanics has evolved, incorporating biophysically-based hyperelastic strain energy laws [2–5], anisotropic tissue structure [6–8], patient-specific geometries [9] and cellular activation [10] to effectively simulate the myocardial behavior assuming basic Newtonian physics [11]. Based on tunable parameters [12,13], cardiac models provide a framework for studying and assessing heart function, offering spatiotemporally varying metrics– such as strain, stress, work and power– which are otherwise inaccessible clinically [14,15].

While cardiac modeling is capable of providing quantitative data of clinical relevance, a number of modeling questions remain actively pursued in the community. An issue commonly discussed in cardiac mechanics is the choice of modeling myocardial tissue as an incompressible [16,3,17,4,18,19,5,20,21] or nearly incompressible [14,22–24] material. While this choice is inherently based on tissue behavior which must be determined experimentally, both models continue to be used either to model incompressible/nearly incompressible behavior or, in some cases, for numerical convenience.

A range of relevant numerical schemes have been applied in heart models, one of the most popular being the penalty method [25,14,22,15,23]. An advantage of this approach is its simplified form, requiring only the solution of the tissue displacement. However, when applied in the finite element method (FEM) framework, displacement-based formulations near the incompressible limit exhibit locking leading to sub-optimal convergence rates and poor numerical approximations in classic elastic models [26–30]. Critically, the penalty method lacks monotonic convergence to the incompressible solution as the bulk modulus is increased, making it challenging to employ as an approximate model to an incompressible cardiac material model.

The development of numerical strategies circumventing these issues has been a field of significant research effort in the solid mechanics community. Among others, the B-Bar method introduced by Hughes [31] and its generalization to finite strains [32,33], the reduced or selective integration technique [34,30,35], the augmented Lagrangian method [36,37], have been successfully employed to enforce incompressibility while tackling the numerical difficulties and locking phenomena associated with the penalty formulation. An alternative approach used extensively in solid mechanics, also known to alleviate locking, is the class of multi-field variational principles, which gained popularity with the pioneering work of Herrmann on isotropic linear elasticity [38]. Herrmann’s principle was also extended to orthotropic materials by Taylor [39] and Key [40], to nonlinear formulations [41,42] and elasto-plastic applications [43].

The most common of these mixed formulations is the Lagrange Multiplier (LM) method, a two-field variational approach which has been used widely to enforce incompressibility of the myocardium by introducing a variable to respresent the hydrostatic pressure [16,17,4,19,44]. While the LM method is known to improve numerical convergence [45,46,29,47] and avoid locking phenomena, the use of an additional variable results in increased computational cost and enhanced complexity in the linear algebra involved, due to the indefinite nature of the resulting stiffness matrix [26,45].

The Perturbed Lagrangian (PL) formulation was introduced to address this issue, by augmenting the energy functional of the LM approach with a penalty/compressibility term [48–50]. The PL is a two-field variational approach suitable for the solution of nearly incompressible problems, where pressure and displacement are treated as independent variables. Sussman and Bathe introduced a generalized form of the PL approach, the u/p formulation, which has been used extensively in the computational mechanics literature [51,52,48] and has also been applied in the myocardium [44]. Similarly, the well established three-field Hu-Washizu formulation by Simo et al. [33] extends the PL formulation by introducing pressure and dilatation as independent variables [37,46,51,53]. This approach has also been employed in cardiac mechanics [24] (though this procedure comes with the cost of computing an additional variable). The use of a separate interpolation for the independent variables, allows efficient and accurate approximations, alleviating the numerical difficulties associated with both the penalty and LM methods. The efficiency of these methods was also enhanced with the use of discontinuous interpolation for the pressure and dilatation fields (static condensation) [50,47,33,46] allowing the estimation of these fields on element level and leading to a generalized displacement-only formulation. Further, Bercovier [50] proved that, for Herrmann’s principle, the PL (and its statically condensed equivalent) converges monotonically to the incompressible problem as the bulk modulus is increased. Nevertheless, as suggested by Sussman and Bathe [47], static condensation may exhibit convergence difficulties during the Newton–Raphson procedure.

In this paper, we consider the statically condensed Perturbed Lagrangian formulation of Bercovier [50] and others [48,49], which may be conveniently thought of as a weakly penalized form with an optional choice of projection operator. In this generalized form, with an appropriate choice of the projection operator, we may choose to strengthen or weaken the constraint resulting in the PL, LM or penalty formulations. Using this generalization, we derive an estimate detailing the error convergence of these methods (in a linear setting) and introduce modifications to a Newton-Raphson scheme [54,55] to significantly improve nonlinear convergence properties for standard and weakly penalized formulations (particularly for high bulk modulus). The scheme is further augmented to take advantage of a Shamanskii-type Newton scheme [54,55] boosting computational performance by enabling re-use of the Jacobian matrix (and its inverses or preconditioners) estimated at previous time/loading steps. As this re-use is particularly sensitive to stiffness, we modify the scheme to effectively maintain nonlinear convergence behavior. Further, we examine the direct numerical discretization of the weakly penalized form which may be made efficient through the use of discontinuous projection operators. The weakly penalized form is then compared with the mixed variational formulations (LM, PL), as well as the penalty method, showing that the modified form maintains the convergence characteristics of the mixed variational forms and avoids locking behaviors observed in the penalty method. This comparison is performed on a model left ventricle, which to the best of our knowledge is the first application of this combination of the PL method and static condensation in cardiac mechanics. Further we verify the result proven for linear problems in [50], showing that the error between the weakly penalized formulation and the incompressible solution indeed decreases with a rate inversely proportional to the bulk modulus. As a result, the formulation enables modeling of the myocardium as nearly incompressible or incompressible (with an error proportional to 1/k, with *k* being the bulk modulus).

Below we expand on this approach to illustrate the general minimization problem (Section 2.1) and show how both penalty and LM formulations may be thought of equivalently as weakly penalized constraints in the continuous setting (Section 2.1.1). The basis for locking is then reviewed in Section 2.2, motivating the introduction of the weakly penalized approach. The different convergence behavior of the various schemes is also illustrated through their error estimates at the solution of a linear incompressible problem (Section 2.4). We then introduce modifications to the mechanical system to improve the nonlinear convergence behavior of the weakly penalized scheme for high values of the bulk modulus (Section 2.5.3). Moreover, we modify the SNR scheme for the weakly penalized and penalty formulations to enable better computational efficiency (Section 2.5.4). The numerical convergence of these different methods is then compared, showing optimal convergence and locking phenomena in the various schemes [27,56] for a two-dimensional problem and a cardiac model (Section 3). Finally, the LM, penalty and weakly penalized formulations are compared in terms of accuracy and convergence for whole-cycle cardiac mechanics. Our results suggest that the weakly penalized form provides an accurate and computationally efficient alternative to the LM, PL and penalty methods and can be applied successfully in the numerical implementation of incompressible and nearly incompressible cardiac models (Section 4).

## 2 Methods

In this section we show how LM and Penalty formulations can be viewed uniformly through a weakly penalized form (PL) (Section 2.1). Subsequently, we introduce the discretized forms, illustrating the deviation of the two schemes and resulting locking phenomena (Section 2.2). These motivate the use of an alternative discretization strategy, leading to a displacement-only formulation. The solution to this system is then demonstrated and optimized to accommodate the numerical stiffening due to the weakly penalized terms.

### 2.1 Continuous minimization problem

**Fig. 1:**
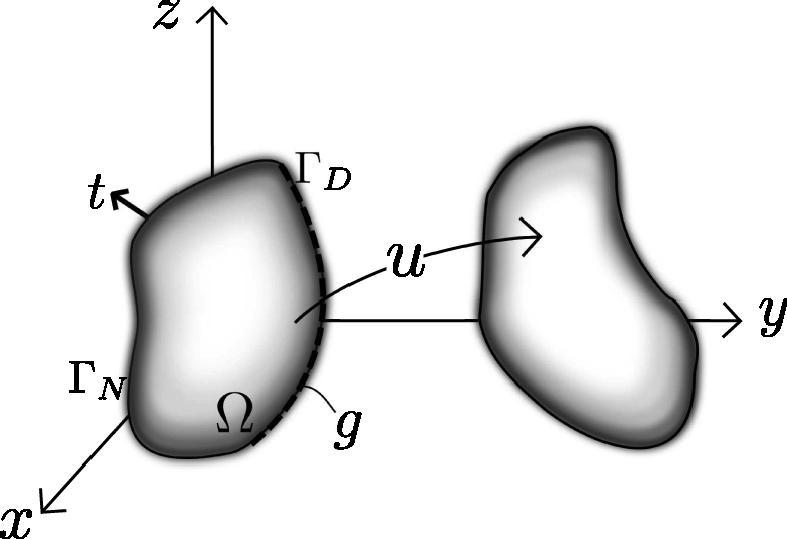
The undeformed and deformed body under consideration. Here Ω represents the reference state of the body, $\Gamma =\Gamma_{D}\cup \Gamma_{N}$ its boundaries subject to Dirichlet and traction conditions, respectively.

Problems of static (or quasi-static) solid mechanics involve finding the deformation, u, of a body defined over a domain Ω as shown in Fig. 1. Here, the body is under the action of a body force, $f:\Omega \to R^{d}\text{,}d⩾1$, and some boundary traction $t:\Gamma_{N}\to R^{d}$.

The solution u is a function commonly sought in an appropriate function space X (usually $X\subset H^{1}(\Omega )$ or a space smooth enough to ensure existence and uniqueness of the solution of the minimization problem [61,48]), subject to the Dirichlet boundary condition $u_{{|}_{\Gamma_{D}}}=g$, *i.e.*

$$X_{D}=\{v\in X|v_{{|}_{\Gamma_{D}}}=g\}\text{.}$$

The displacement of the body may be obtained by the principle of virtual work, equivalent to the principle of stationary potential energy [45]. Following the principle of stationary potential energy we seek to find a minimizer of the total potential energy functional, $\Pi :X_{D}\to R$, describing the total potential energy of the body under consideration (see Fig. 1). Assuming that the traction and body forces are not functions of the displacement u, then under static equilibrium, the total potential energy for a hyperelastic body may be expressed as a sum of the internal and external potential energy as,

(1) $$\Pi (v)=\Pi_{\mathrm{int}}(v)+\Pi_{\mathrm{ext}}(v)\text{,}$$

(2) $$\Pi_{\mathrm{int}}(v)=\int_{\Omega }\Psi (v)\mathrm{dV}\text{,}\;\Pi_{\mathrm{ext}}(v)=-\int_{\Omega }f\cdot v\mathrm{dV}-\int_{\partial \Omega }t\cdot v\mathrm{dA}\text{,}$$

where $\Psi :X\to R^{+}$ represents the strain energy function [45]. According to the principle of stationary potential energy, the body will deform in a way that minimizes its total potential energy Π. This problem can be expressed as,

(3) $$\Pi (u)=\underset{v\in X_{D}}{\inf}\Pi (v)\text{.}$$

In the case of incompressibility, the deformation is required to preserve the determinant of the deformation gradient $F_{v}$,

$$J_{v}-1=0\text{,}\;J_{v}=|F_{v}|=|\nabla v+I|\text{.}$$

In this case, the solution is found [29], which satisfies,

(4) $$\Pi (u)=\underset{v\in X_{J}}{\inf}\Pi (v)\text{,}$$

(5) $$X_{J}=\{v\in X_{D}|J_{v}-1=0\text{,}a.e.\;\mathrm{on}\;\Omega \}\text{.}$$

We note that as it is not, in general, straightforward to construct the space $X_{J}$ it is often preferable to seek the solution in the entire $X_{D}$ space.

#### 2.1.1 Weakly penalized form and the penalty/LM/PL methods

For later comparisons, in this section we introduce a weakly penalized form of the mechanical problem and show its equivalence with both penalty and LM formulations. Here we introduce the projection operator $\pi_{W}:L^{2}(\Omega )\to W$ which, for any function $g\in L^{2}(\Omega )$, denotes the orthogonal projection onto *W*, *i.e.*

(6) $$(g-\pi_{W}(g)\text{,}q)≔\int_{\Omega }[g-\pi_{W}(g)]\mathrm{qdV}≔0\text{,}\;\forall q\in W\text{.}$$

In this way, we may elect to represent *g* coarsely or finely by adjusting the selection of the space *W* (as we will discuss further in the following sections). We may then introduce the weakly penalized total potential energy functional,

(7) $$\Pi_{P}(v)=\int_{\Omega }\Psi (v)+\frac{1}{2}k{\left[\pi_{W}(J_{v}-1)\right]}^{2}\mathrm{dV}+\Pi_{\mathrm{ext}}(v)\text{,}$$

where an additional penalty term has been added, representing the growth in energy resulting from material compression as is typical for many penalty methods. However, the presence of the projection $\pi_{W}$ enables the selective weakening or strengthening of the constraint by allowing it to hold weakly through Eq. (6). Clearly, when $J_{v}\in W$ for any v∈X (for example, when $W≔L^{2}(\Omega )$ as is the case for the continuous mechanical system [48]), then

(8) $$\pi_{W}(J_{v}-1)=J_{v}-1$$

and Eq. (7) reduces to the classic total potential energy functional,

(9) $$\Pi_{k}(v)=\int_{\Omega }\Psi (v)+\frac{1}{2}k{\left(J_{v}-1\right)}^{2}\mathrm{dV}+\Pi_{\mathrm{ext}}(v)\text{,}$$

where the *k*-dependent term denotes the volumetric penalty term often used in cardiac mechanics [25,23]. The Perturbed Lagrangian formulation may be derived by introducing an additional variable, λ∈W with,

(10) $$\lambda ≔k\pi_{W}^{v}\text{.}$$

Substituting the orthogonal projection with the added variable, and adding the Galerkin orthogonality condition (Eq. (6)) we arrive at the PL functional [50,47,48,57],

(11) $$\Pi_{\lambda }(v\text{,}\lambda )=\int_{\Omega }\Psi (v)+\lambda (J-1)-\frac{\lambda^{2}}{2k}\mathrm{dV}+\Pi_{\mathrm{ext}}(v)\text{.}$$

This general purpose formulation has been employed for the solution of nearly incompressible materials [47,50,48,49] and cardiac mechanics [44]. Note that as k→∞ the previous formulation becomes the classic LM method [26,29].

In the continuous setting, there is a solution u∈X which satisfies,

(12) $$\Pi_{P}(u)≔\underset{v\in X}{\inf}\Pi_{P}(v)\text{,}$$

(13) $$\Pi_{k}(u)≔\underset{v\in X}{\inf}\Pi_{k}(v)\text{,}$$

(14) $$\Pi_{\lambda }(u\text{,}\lambda )≔\underset{v\in X}{\inf}\underset{q\in W}{\sup}\Pi_{\lambda }(v\text{,}q)$$

for all approaches and all values of *k*. However, this equivalence is often lost in the discrete setting as different strategies are applied to discretize the function space, X, and the orthogonal projection, $\pi_{W}$.

### 2.2 Finite element approximation

In the FEM framework used in the solution of Problems (12)–(14), the domain Ω is subdivided into a mesh of non-overlapping elements [56]. The displacement is then interpolated with functions in $X^{h}\subset X$, consisting of a set of piecewise polynomials ($P^{k_{u}}$) on the mesh T=T(Ω), i.e.

$$X^{h}≔\{v^{h}\in C(\overset{¯}{\Omega })|\;v^{h}{|}_{\tau }\in P^{k_{u}}\text{,}\;\forall \tau \in T(\Omega )\}\text{,}$$

where $k_{u}$ denotes the order of interpolation used for the displacement and C is the space of continuous vector functions. Letting $\Phi_{u}$ be an $N_{u}$ vector function comprised of the basis functions ${\left\{\Phi_{i}\right\}}_{i=1}^{N_{u}}$, the resulting displacement solution is then expressed as the weighted sum,

(15) $$u^{h}=U\cdot \Phi_{u}\text{,}\;U\in R^{N_{u}}\text{.}$$

In all approaches, the minimization of the total potential energy occurs over $X^{h}\subset X$.

The primary point of departure between the penalty and PL formulations comes in the choice of orthogonal projection. In the case of the penalty method, the orthogonal projection $\pi_{W}:L^{2}(\Omega )\to W$ remains on the continuous space $W=L^{2}(\Omega )$, leaving the total potential energy $\Pi_{k}$ unchanged. In contrast, the PL approach given in Eqs. (11) and (14) requires a numerical approximation of the pressure variable, λ by $\lambda^{h}$, it is hence natural to introduce a discrete function space $W^{h}\subset W$, *i.e.*

$$W^{h}≔\{q^{h}\in C(\overset{¯}{\Omega })|\;q^{h}{|}_{\tau }\in P^{k_{p}}\text{,}\;\forall \tau \in T(\Omega )\}\text{.}$$

This is equivalent to introducing the projection operator $\pi_{W^{h}}:L^{2}(\Omega )\to W^{h}$ which satisfies the Galerkin orthogonality condition (Eq. (6)) on $W^{h}$. This change means that $\pi_{W^{h}}$ projects the incompressibility onto a discrete set of polynomials of degree $k_{p}$, effectively relaxing strict satisfaction of the constraint. The weakening of the constraint through the projection operator is similar to reduced/selective integration techniques which also weaken the constraint on compressibility/incompressibility. However, while these techniques have been proven consistent for specific quadrature and element schemes [26], proof is required to ensure each scheme achieves an optimal rate of convergence. The spaces $X^{h}$ and $W^{h}$ for the weakly penalized approach are often selected to satisfy the inf–sup condition (where we note $k_{p}<k_{u}$), which ensures uniqueness in the multiplier for all *k* [58,26,59–61]. Additionally, for appropriately selected spaces $W^{h}$– such as $Q^{k_{u}}-Q^{k_{p}}$ with $k_{u}=k_{p}+1$– convergence rates in the energy norm are optimal (*i.e.* $O(h^{k_{u}})$).

It is well known that these two methods need not be equivalent in the discrete setting, as can also be confirmed by the presence of locking phenomena in penalty applications [26,27]. These facts can also be observed through the dependence of the methods on the penalty parameter *k*. As k→∞, the PL becomes the classic LM method and the approximation spaces reduce to the subsets,

(16) $$X_{k}^{h}=\{v^{h}\in X^{h}|\pi_{W}(J_{v^{h}}-1)=0\}\text{,}$$

(17) $$X_{\lambda }^{h}=\{v^{h}\in X^{h}|\pi_{W^{h}}(J_{v^{h}}-1)=0\}\text{,}$$

for the penalty and LM methods, respectively. These spaces are nested, *i.e.*

(18) $$X_{k}^{h}\subseteq X_{\lambda }^{h}\subseteq X^{h}\text{,}$$

illustrating the more restrictive subset of functions over which the minimization problem can be considered. As a consequence, the space $X_{k}^{h}$ is typically a small subset of $X^{h}$ and can be too restrictive. This occurs as a small violation of the incompressibility constraint can cause a significant increase in the strain energy even though the approximate solution may have a minor degree of error from the true solution.

In contrast, while the LM approach effectively weakens the satisfaction of the constraint, it also has proven optimal convergence rates when $X^{h}$ and $W^{h}$ are chosen to satisfy the inf–sup condition [26]. This circumvents over-constraining of the approximation space, but comes at the expense of computing an additional variable.

### 2.3 Discrete weakly penalized form

In the discrete setting, the projection operator $\pi_{W^{h}}:L^{2}(\Omega )\to W^{h}$ introduced in Eq. (6) can be written as,

(19) $$(g-\pi_{W}^{h}(g)\text{,}q^{h})≔Q^{T}(R_{g}-M\pi )≔0\text{,}\;\forall q^{h}\in W^{h}\text{,}$$

where $q^{h}=Q\cdot \Phi_{w}$ is a test function in $W^{h}\text{,}\pi_{W}^{h}(g)=\pi \cdot \Phi_{w}$ denotes the projection of *g* on $W^{h}\text{,}\;M$ is the $W^{h}-$ mass matrix,

(20) $${\left[M\right]}_{\mathrm{ij}}≔\int_{\Omega }\phi_{w}^{i}\;\phi_{w}^{j}\mathrm{dV}\text{,}\;\phi_{w}^{i}\text{,}\phi_{w}^{j}\in W^{h}$$

and $R_{g}$ is the weighted function over the test space $W^{h}$,

(21) $${\left[R_{J}\right]}_{j}=\int_{\Omega }\phi_{w}^{j}\mathrm{gdV}\text{,}\;\phi_{w}^{j}\in W^{h}\text{.}$$

Considering the introduced term in Eq. (7), we may write

(22) $$\int_{\Omega }\frac{1}{2}k{\left[\pi_{W}(J_{v}-1)\right]}^{2}\mathrm{dV}=\frac{1}{2}k\pi^{T}M\pi \text{,}$$

where here $\pi \cdot \Phi_{w}=\pi (J_{v^{h}}-1)$. Following from Eq. (19) and noting the requirement that the projection holds for $q^{h}\in W^{h}$ is equivalent to requiring it hold for all $Q\in R^{N_{w}}$ (where $N_{w}$ is the dimension of the discrete space $W^{h}$), π can be seen to satisfy the linear system,

(23) $$M\pi =R_{J}\text{,}$$

where M is given in Eq. (20)

(24) $${\left[R_{J}\right]}_{j}=\int_{\Omega }\phi_{w}^{j}(J_{v}^{h}-1)\mathrm{dV}\text{,}\;\phi_{w}^{j}\in W^{h}\text{.}$$

Inverting M in Eq. (23) and substituting into Eq. (22), the weakly penalized system (Eq. (7)) may be written in discrete form as,

(25) $$\Pi_{P}(v^{h})=\int_{\Omega }\Psi (v^{h})\mathrm{dV}+\frac{1}{2}kR_{J}^{T}M^{-1}R_{J}+\Pi_{\mathrm{ext}}(v^{h})\text{.}$$

This form, also used by Bercovier [50] and others [48,49], reduces the system into a single minimization problem on $X^{h}$, eliminating the pressure variable. However, the presence of $M^{-1}$ requires the solve of Eq. (23), incuring similar computational cost as computing the PL solution. Considering the discrete weak form and its solution (as we show later), this weakly penalized term requires matrix–matrix products which are (1) expensive, (2) nonlinearly dependent on the solution and (3) generally of a more dense sparsity than the standard penalty system.

These practical issues presented stem from the choice of global orthogonal projection, $\pi_{W^{h}}$, which unfortunately complicates the computation. However, the choice of $\pi_{W^{h}}$ is, generally speaking, arbitrary and ideally should balance the need for accuracy with ease of computation. An alternative approach is to use a local orthogonal projection, $\pi_{W_{\mathrm{loc}}^{h}}$, satisfying

(26) $$M_{\tau }\pi_{l}=R_{J\text{,}\tau }\text{,}\;\forall \tau \in T\text{,}$$

where $M_{\tau }$ and $R_{J\text{,}\tau }$ are the mass matrix and the weighted constraint vectors on the element τ. That is, the local orthogonal projection, $\pi_{W_{\mathrm{loc}}^{h}}$, satisfies Eq. (6) on the piecewise discontinuous space,

$$W_{\mathrm{loc}}^{h}≔\{q^{h}\in L^{2}(\Omega )|\;q^{h}{|}_{\tau }\in P^{k_{\pi }}\text{,}\;\forall \tau \in T(\Omega )\}\text{.}$$

Using this locally continuous, but globally discontinuous interpolation space, the total potential energy for the body becomes,

(27) $$\Pi_{P}(v^{h})=\int_{\Omega }\Psi (v^{h})\mathrm{dV}+\frac{k}{2}\underset{\tau \in T}{\sum }R_{J\text{,}\tau }^{T}M_{\tau }^{-1}R_{J\text{,}\tau }+\Pi_{\mathrm{ext}}(v^{h})\text{.}$$

This localized projection, also known as static condensation has been employed by Sussman and Bathe [47], Bercovier [50] and Simo et al. [33,37] to enhance the efficiency of the formulation, while avoiding over-constraining of the approximation space and locking phenomena. Indeed, by localizing the projection, computations remain on the element level, reducing the computational cost relative to Eq. (25). Moreover, localization of the penalty term preserves sparsity of the penalty system, significantly reducing sparsity to that resulting from Eq. (25). These practical improvements come at the cost of restricting the approximation space. Again, as we send k→∞, the approximation space of the weakly penalized formulation in Eq. (27) is restricted to the space,

$$X_{P}^{h}=\{v^{h}\in X^{h}|\pi_{W_{\mathrm{loc}}^{h}}^{v^{h}}=0\}\text{.}$$

which we note is,

$$X_{k}^{h}\subseteq X_{P}^{h}\subseteq X_{\lambda }^{h}\text{.}$$

Note that in a practical setting, as *k* can not be infinite, an augmented Lagrangian iterative scheme can be applied to iteratively increase *k*. In this way, the weakly penalized form can provide equivalent results to the incompressible LM method.

Though the weakly penalized form in Eq. (27) does not mandate the inf–sup condition, usage of inf–sup stable spaces (such as Nicolaides–Boland [60] or Crouzeix–Raviart [62] elements) with globally discontinuous pressure ensures optimal convergence. However, as we demonstrate, even for some spaces which are not inf–sup stable (for instance $Q^{2}-Q_{\mathrm{loc}}^{1}$), this weakening of the constraint is sufficient to restore convergence.^1^

### 2.4 Error estimates for the generalized weakly penalized form

Using the generalized weakly penalized form, Appendix B shows an error estimate in Lemma 1 in the case of a linear elastic model. Here, the projection and bulk modulus in the discrete model $\left(\pi^{h}\text{,}k_{h}\right)$ are left general, enabling extension to the methods (LM, PL, and Penalty) considered in the paper. From this analysis, we observe in Lemma 1 (when $k_{h}=k$) that the continuous model ($u=u^{*}+(1/k)u^{⊥}$) and discrete model satisfy the estimate,

(28) ‖u-uh‖1⩽Cinfyh∈X0,divhzh∈(X0,divh)⊥‖πh(∇·u⊥)-∇·u⊥‖+‖u∗-yh‖1+1k+1‖u⊥-zh‖1,

(see Appendix B). Here, the estimate shows the approximation depends on three principle terms which relate the error incurred due to the projection as well as the error approximating divergence free and non-divergence free components of the solution. Importantly, the bound depends on the subspaces $X_{0\text{,}\mathrm{div}}^{h}$ and ${\left(X_{0\text{,}\mathrm{div}}^{h}\right)}^{⊥}$ which, in general do not exhibit straightforward convergence properties. However, as demonstrated in Corollary 1 of Appendix B, if $\pi^{h}$ projects $L^{2}(\Omega )$ to a discrete space which satisfies the inf–sup condition [50,59,26,61], the estimate can be extended so the infimum is taken over $X_{0}^{h}$. This factor – which is exploited in the LM and PL formulations (as well as the weakly penalized form suggested above) – enables straightforward application of standard estimates derived from interpolation theory.

However, in the case of the penalty method, $\pi :L^{2}(\Omega )\to L^{2}(\Omega )$ remains the continuous $L^{2}$-projection, limiting the extension discussed. While some simplification can be made (see Corollary 2), the estimate requires use of $X_{0\text{,}\mathrm{div}}^{h}$ which may be prohibitive, limiting the convergence behavior. While more straightforward estimates may be derived (see for example [36]), in these the scaling constant *C* depends on *k*.

These results are in agreement with the the numerical results presented later in this paper.

### 2.5 Discrete weak form of the weakly penalized formulation

This section deals with the weak form of the weakly penalized formulation and the modifications introduced in the residual evaluation to enable improved nonlinear convergence and better performance of the SNR scheme. The discrete weak form for the weakly penalized formulation can be obtained by requiring that the directional derivative of the total potential energy functional vanishes in all arbitrary directions $\delta u^{h}\in X_{0}^{h}$ at $u^{h}$, *i.e.*

(29) $$D\Pi_{P}(u^{h})[\delta u^{h}]=0\text{,}\;\forall \delta u^{h}\in X_{0}^{h}\text{,}$$

with $X_{0}^{h}$ the homogeneous zero Dirichlet subspace of $X^{h}$. Following this procedure, the discrete weak form can be written in operator notation as,

(30) $$R(u^{h}\text{,}\delta u^{h})≔A(u^{h}\text{,}\delta u^{h})+C(u^{h}\text{,}\delta u^{h})-F(\delta u^{h})=0\text{,}$$

where *R* is the residual function and the operators A,F, and *C* are defined as,

$$\begin{array}{cc} & A(u^{h}\text{,}\delta u^{h})=\int_{\Omega }F_{h}S_{h}:\nabla \delta u^{h}\mathrm{dV}\text{,}\\ & F(\delta u^{h})=\int_{\Omega }f\cdot \delta u^{h}\mathrm{dV}+\int_{\partial \Omega }t\cdot \delta u^{h}\mathrm{dA}\text{,}\\ & C(u^{h}\text{,}\delta u^{h})=k\underset{\tau \in T}{\sum }\delta U_{\tau }^{T}B_{\tau }^{T}M_{\tau }^{-1}R_{J\text{,}\tau }\text{,} \end{array}$$

where $F_{h}=\nabla u^{h}+I$ and $S_{h}$ are the discrete deformation gradient and second Piola stress tensors, respectively [45]. Here $\delta U_{\tau }$ represents the local basis coefficients for $\delta u^{h}$ on the element and the element matrix $B_{\tau }$ denotes the linearized constraint derived from the PL functional, *i.e.*

(31) $${\left[B_{\tau }\right]}_{\mathrm{ij}}=B_{\tau }(\phi_{w}^{i}\text{,}u^{h}\text{,}\phi_{u}^{j})=\int_{\tau }\phi_{w}^{i}J_{u^{h}}F_{h}^{-T}:\nabla \phi_{u}^{j}\mathrm{dV}\text{,}$$

with $(\phi_{w}^{i}\text{,}\phi_{u}^{j})\in W_{\mathrm{loc}}^{h}\times X^{h}$. Note that $M_{\tau }$ and $R_{J\text{,}\tau }$ are identical to those in Eq. (20) and (24) with $\phi_{w}^{i}\in W_{\mathrm{loc}}^{h}$.

The weak forms for the penalty, LM and PL formulations can be derived similarly as outlined in Appendix A.

#### 2.5.1 Nonlinear solution for the weakly penalized problem

In order to solve the mechanical system introduced in Eq. (30) (as well as the others discussed in the Appendix A), we look to use the global Shamanskii–Newton–Raphson (SNR) method [54]. This method has been shown to be effective for problems in fluid–structure interaction [55], enabling faster computation by re-using the Jacobian matrix over multiple time/load steps. Following the procedure outlined in [55], on the $n^{\text{th}}$ SNR iteration we update each subsequent guess of the solution, $u^{h}=U^{n}\cdot \Phi_{u}$, using the iterative formula,

(32) $$U^{n+1}=U^{n}+\delta U^{n}\text{,}\;\delta U^{n}=-\alpha^{n}{\left[J(U^{\beta })\right]}^{-1}R(U^{n})\text{.}$$

Here $U^{n}$ denotes the basis weights at the $n^{\mathrm{th}}$ iteration, $\delta U^{n}$ the update vector, and R and J are the residual and Jacobian, respectively, defined in Eq. (33).

(33) $${\left[R(U^{n})\right]}_{i}=R(U^{n}\cdot \phi_{u}\text{,}\phi_{u}^{i})\text{,}\;{\left[J(U^{n})\right]}_{\mathrm{ij}}\approx \frac{\partial R(U^{n}\cdot \phi_{u}\text{,}\phi_{u}^{i})}{\partial U_{j}^{n}}\text{.}$$

The key distinction between the Newton–Raphson method, global Newton–Raphson method and SNR method introduced in Eq. (32) are the parameters $\alpha^{n}$ and β. In classical Newton–Raphson, these parameters play no role in the update process (in this case $\left(\alpha^{n}\text{,}\beta )=(1\text{,}n\right)$). The global Newton–Raphson scheme, however, uses the parameter $\alpha^{n}\in [0\text{,}1]$ to scale the descent direction to minimize $\left\|R(U^{n+1})\right\|$ and ensure that $\left\|R(U^{n+1})\|⩽\|R(U^{n})\right\|$, *i.e.* that the residual decays (in this case β=n).

**Table:** 

**Algorithm 1.** Shamanskii–Newton–Raphson (SNR)
Given initial guess $U^{0}\text{,}\beta$, and $S_{0}$. Compute $R(U^{0})$, and set n=0.
*while*$S_{n}\|R(U^{n})\|>\mathrm{TOL}$
*if* (β=n) {*Compute*$J(U^{n})$*and*${\left[J(U^{n})\right]}^{-1}$ (or preconditioner)}
*Solve*$J(U^{\beta })\delta U^{\prime }=-R(U^{n})$.
*Find*$\min\;\{\;\|R(U^{n}+\alpha^{n}\delta U^{\prime })\|\text{,}\;\alpha^{n}\in [0\text{,}1]\}$.
*Set*$\delta U^{n}=\alpha^{n}U^{\prime }\text{,}U^{n+1}=U^{n}+\delta U^{n}$.
*if* ($\left\|R(U^{n+1})\|>\gamma S_{n}\|R(U^{n})\right\|$ *or*n> ITER) {*Set*β=n+1}
*Set*n=n+1, *and*$S_{n}=1$
*end* *while*

In constrast, the SNR method shown in Algorithm 1 combines the minimization procedure with a re-use scheme, where β⩽n denotes the use of a Jacobian matrix determined at a previous load step/iteration. The selection of β is based on the convergence characteristics governed by $\gamma \text{,}S_{n}$ and *ITER*.^2^ Here γ∈(0,1] governs the degree of residual decrease required to continue using the currently stored Jacobian. *ITER* may also signal re-computation and enables a cap on the degree of re-use. Finally, the parameters $S_{0}$ is used to cope with stiff problems which, due to Dirichlet conditions, may see initial increases in the residual computation.

#### 2.5.2 Jacobian and residual construction for the weakly penalized problem

As both penalty and LM formulations have been outlined elsewhere, here we focus on the Jacobian and residual evaluations for the weakly penalized approach. Note that $J\in R^{N_{u}\times N_{u}}$ and $R\in R^{N_{u}}$ may be written as,

(34) $$J(U^{\beta })=AA_{\tau }(U^{\beta })+C_{\tau }(U^{\beta })\text{,}$$

(35) $$R(U^{n})=AR_{A\text{,}\tau }(U^{n})+R_{P\text{,}\tau }(U^{n})\text{,}$$

where A is the FEM assembly operator, τ∈T denotes specific elements, the subscript τ denotes vector, matrices or operators constructed on the element and $R_{A\text{,}\tau }$ and $R_{P\text{,}\tau }$ are element-level residual contributions stemming from the weakly penalized term, $R_{P}$, and all other terms, $R_{A}$. Introducing a short-hand notation, i.e. $C_{\tau }^{\beta }=C_{\tau }(U^{\beta })$, we can express the element-level Jacobian contributions $A_{\tau }$ and $C_{\tau }$ as,

(36) $${\left[A_{\tau }^{\beta }\right]}_{\mathrm{ij}}=\frac{1}{∊}\left[A_{\tau }(u_{\beta }^{h}+∊\phi_{u}^{j}\text{,}\phi_{i})-F_{\tau }(u_{\beta }^{h}+∊\phi_{u}^{j}\text{,}\phi_{i})-A_{\tau }(u_{\beta }^{h}-∊\phi_{u}^{j}\text{,}\phi_{i})+F_{\tau }(u_{\beta }^{h}-∊\phi_{u}^{j}\text{,}\phi_{i})\right]\text{,}$$

(37) $$C_{\tau }^{\beta }=k{\left[B_{\tau }^{\beta }\right]}^{T}M_{\tau }^{-1}B_{\tau }^{\beta }\text{,}$$

(38) $${\left[R_{\tau }^{n}\right]}_{j}=A_{\tau }(u_{n}^{h}\text{,}\phi_{u}^{j})-F_{\tau }(\phi_{u}^{j})\text{,}$$

(39) $$R_{P\text{,}\tau }^{n}=k{\left[B_{\tau }^{n}\right]}^{T}M_{\tau }^{-1}R_{J\text{,}\tau }^{n}\text{,}$$

The element-level matrix $A_{\tau }$ denotes those terms resulting from the elasticity stress/boundary contributions^3^ and is evaluated using central finite differencing (typically $∊=10^{-4}h$, where *h* is the mesh size). $C_{\tau }$ denotes those terms which result from the weakly penalized form. Here we assume that $B_{\tau }$, defined in Eq. (31), is independent of u when we linearize the *C* operator introduced in Section 2.5. This linearization does not seem to impact convergence of the Newton scheme and preserves symmetric positive semi-definiteness of the weakly penalized matrix term. As we see, $C_{\tau }$ is comprised of the local element mass matrix $M_{\tau }$ and the linearized constraint equation $B_{\tau }$ introduced in the previous section. We note that $M_{\tau }$ and its inverse are linear and thus may be computed once for the entire simulation. On the other hand, the linearized constraint must be re-computed due to its nonlinear dependence on the solution. However, computing this matrix is quick as it does not require differencing.

#### 2.5.3 Residual modifications for the nonlinear solve of the weakly penalized system

As mentioned previously, the weakly penalized system can be thought of as a generalized formulation which can result in the PL, penalty or statically condensed PL formulations depending on the choice of the projection operator. The equivalence of these methods under the weakly penalized regime, allows us to combine and take advantage of the good characteristics of each method. For instance, the weakly penalized formulation combines the simplified structure of the penalty method with the convergence characteristics of the PL formulation. However, due to the stiffness of the linear system at high values of the bulk modulus, the penalized formulations (classic penalty/weakly penalized) exhibit deteriorated nonlinear convergence. This stands in stark contrast to the PL method which (for inf–sup stable schemes) exhibits fast convergence even for high bulk modulus. However, we observe that, when the choice of $\pi^{h}$ provides equivalence with the discrete PL method, poor nonlinear convergence is observed though, in principle, the convergence should be similar. Examining the update formulae for both weakly penalized and PL approaches (see Appendix C), we observe that deteriorated convergence stems from: (1) initial residual amplification, and (2) the amplification of the residual.

The first factor, mentioned in Section 2.5.1, results from non-monotonicity in the residual. This manifests particularly early during the nonlinear solve, where the initial residual becomes amplified after the first iteration. This is particularly evident with Dirichlet conditions on a stiff material, where the norm of the boundary displacement (for example) may be much smaller than the updated residual due to stiffness in the material. This issue which is not observed in the PL solution, is circumvented in the Newton–Raphson procedure and the SNR procedure outlined in Algorithm 1 by enabling an initial amplification of the residual and relaxing strict requirements on monotonicity.

Less trivial issue to address is the amplification of the residual resulting from large *k*, which can lead to poor convergence or stalling in the iterative solve. This increased residual, due to strong dependence on the weakly penalized term, often does not imply divergence but rather results from a *k*-dependent scaling of the projection problem and its nonlinear dependence on $u^{h}$ as shown in Appendix C. Based on Eq. (C.7) (and choosing β=n), we may re-write the weakly penalized residual contribution in terms of the linearized guess and a remainder,

(40) $$R_{P\text{,}\tau }^{n}=R_{P^{*}\text{,}\tau }+k{\left[B_{\tau }^{n}\right]}^{T}R_{e\text{,}\tau }\text{,}$$

(41) $$R_{P^{*}\text{,}\tau }=k{\left[B_{\tau }^{n}\right]}^{T}M_{\tau }^{-1}(R_{J\text{,}\tau }^{n-1}+B_{\tau }^{n-1}\delta U_{\tau }^{n-1})\text{,}$$

(42) $$R_{e\text{,}\tau }=M_{\tau }^{-1}(R_{J\text{,}\tau }^{n}-R_{J\text{,}\tau }^{n-1}-B_{\tau }^{n-1}\delta U_{\tau }^{n-1})\text{.}$$

Here the first term approximates the current linearized guess of the projection, while the second examines how well the current guess satisfies the projection problem. In other words, the first term denotes the hydrostatic contribution to momentum, while the second represents the error remaining in the projection problem amplified by the bulk modulus *k*. For large *k*, the later term can become disproportionately scaled, making nonlinear convergence more challenging. Moreover, this issue is avoided in the PL formulation where the hydrostatic constraint is not scaled by *k* as can be seen in the residual terms of Eq. (C.3).

To circumvent this, we modify the Newton–Raphson scheme to measure convergence of $\left\|R+R_{P^{*}}\right\|$ instead of $\left\|R+R_{P}\right\|$. Clearly, $\|R_{P^{*}\text{,}\tau }^{n}-R_{P\text{,}\tau }^{n}\|\to 0$ as $\|\delta U_{\tau }^{n}\|\to 0$; however, measuring convergence of $R_{P^{*}}$ avoids issues due to high bulk modulus. Further, extending this form to the Penalty formulation, we must select the projection $\pi^{h}:L^{2}(\Omega )\to L^{2}(\Omega )$. As, in this case, the rank of M is no longer finite dimensional, we may instead write the modified Penalty form in its equivalent integral form, *i.e.*

(43) $$R_{P\text{,}\tau }^{n}=R_{P^{*}\text{,}\tau }+R_{e\text{,}\tau }\text{,}$$

(44) $${\left(R_{P^{*}\text{,}\tau }\right)}_{i}=\int_{\Omega }k(J^{n-1}+J^{n-1}{\left(F_{h}^{n-1}\right)}^{-T}:\nabla \delta u^{h\text{,}n-1})J^{n}{\left(F_{h}^{n}\right)}^{-T}:\nabla \phi_{i}\mathrm{dV}\text{,}$$

(45) $${\left(R_{e\text{,}\tau }\right)}_{i}=\int_{\Omega }k(J^{n}-J^{n-1}-J^{n-1}{\left(F_{h}^{n-1}\right)}^{-T}:\nabla \delta u^{h\text{,}n-1})J^{n}{\left(F_{h}^{n}\right)}^{-T}:\nabla \phi_{i}\mathrm{dV}\text{.}$$

Similarly to the modifications introduced in the weakly penalized approach, in the modified penalty formulation we measure the convergence of $\left\|R+R_{P^{*}}\right\|$ instead of $\left\|R+R_{P}\right\|$. This avoids the amplification of the error in the second term of the residual, allowing better nonlinear convergence of the scheme.

#### 2.5.4 Residual modifications for the SNR solve of the weakly penalized system

While the Shamanskii–Newton–Raphson scheme can significantly enhance performance of the PL scheme, acceleration in the SNR approach for penalized methods is minimal or even worse than standard Global Newton–Raphson. This deterioration in performance is due, predominantly, to the stiffness of the system for high *k* and the inevitable inaccuracies introduced in the descent direction by the re-used Jacobian. In contrast, this deterioration in performance is not observed in both PL and LM formulations, which can take significant advantage of matrix re-use (as we will show). Once more, by examining the equivalence between weakly penalized and PL formulations (see Appendix C), we observe this deterioration may be circumvented using the modified form,

(46) $$R_{P\text{,}\tau }^{n}=R_{P^{*}\text{,}\tau }+k{\left[B_{\tau }^{\beta }\right]}^{T}R_{e\text{,}\tau }\text{,}$$

(47) $$R_{P^{*}\text{,}\tau }=k{\left[B_{\tau }^{n}\right]}^{T}M_{\tau }^{-1}(R_{J\text{,}\tau }^{n-1}+B_{\tau }^{\beta }\delta U_{\tau }^{n-1})\text{,}$$

(48) $$R_{e\text{,}\tau }=M_{\tau }^{-1}(R_{J\text{,}\tau }^{n}-R_{J\text{,}\tau }^{n-1}-B_{\tau }^{\beta }\delta U_{\tau }^{n-1})\text{.}$$

Note that, as $\|\delta U^{n}\|\to 0$, the difference in the constraint residual $\|R_{J\text{,}\tau }^{n}-R_{J\text{,}\tau }^{n-1}\|\to 0$ and as a result,

$$R_{P\text{,}\tau }^{n}\to k{\left[B_{\tau }^{n}\right]}^{T}M_{\tau }^{-1}R_{J\text{,}\tau }^{n}\text{,}$$

which represents the standard residual resulting from Eq. (30). That is, as we converge, the modified residual $R_{P\text{,}\tau }$ converges to that given by evaluating *C*. Further, we note that if β=n, the first term in the definition $R_{P\text{,}\tau }$ drops away, leaving us with the expected residual.

The modifications we introduced to the residual of the weakly penalized formulation can also be applied in the penalty method to improve its nonlinear convergence behavior by once more selecting the projection operator $\pi^{h}:L^{2}(\Omega )\to L^{2}(\Omega )$ and using the equivalence of the weakly penalized/penalty forms. Expressing these modifications in integral form, we may write the modified Penalty form with residuals,

(49) $$R_{k\text{,}\tau }^{n}=R_{k^{*}\text{,}\tau }+R_{\mathrm{ek}\text{,}\tau }\text{,}$$

(50) $${\left(R_{P^{*}\text{,}\tau }\right)}_{i}=\int_{\Omega }k(J^{n-1}+J^{\beta }{\left(F_{h}^{\beta }\right)}^{-T}:\nabla \delta u^{h\text{,}n-1})J^{n}{\left(F_{h}^{n}\right)}^{-T}:\nabla \phi_{i}\mathrm{dV}\text{,}$$

(51) $${\left(R_{e\text{,}\tau }\right)}_{i}=\int_{\Omega }k(J^{n}-J^{n-1}-J^{\beta }{\left(F_{h}^{\beta }\right)}^{-T}:\nabla \delta u^{h\text{,}n-1})J^{\beta }{\left(F_{h}^{\beta }\right)}^{-T}:\nabla \phi_{i}\mathrm{dV}\text{.}$$

With the introduced modifications in the residual monitored for convergence, the modified penalty method is able to significantly exploit matrix re-use, and substantially improve its computational efficiency.

## 3 Results

In this section we study the convergence behavior of the LM, PL, penalty and weakly penalized formulations outlined in Section 2, in the solution of incompressible mechanics problems, for varying values of *k*. Furthermore, the PL, penalty and weakly penalized approaches are compared on nearly-incompressible solid mechanics problems, assuming various values for the bulk modulus.

### 3.1 Mechanical tests

#### 3.1.1 Elongation of a two-dimensional square domain

**Fig. 2:**
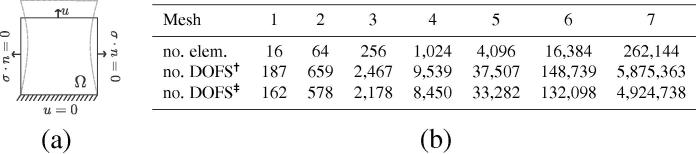
Discretization of the two-dimensional square domain: (*a*) A Neohookean material in a square domain (1×1) under no slip (bottom edge), no traction (side walls), and vertical displacement of 20% (top edge). The shear modulus of μ=100Pa was used. (*b*) Number of elements and degrees of freedom (DOFS) in each discretization for the (†) LM/PL and (‡) weakly penalized/penalty methods.

The convergence behavior of the LM, penalty and weakly penalized methods was compared on the simple case of the stretch of a square domain (Fig. 2(a)). The body was assumed to be made of a Neo-Hookean material, described by the deviatoric strain energy/Second-Piola stress tensor [45],

(52) $$\Psi (C)=\frac{\mu }{2}\left(\frac{I_{C}}{{\mathrm{III}}_{C}^{1/d}}-d\right)\text{,}\;S=\frac{\mu }{{\mathrm{III}}_{C}^{1/d}}\left(I-\frac{I_{C}}{d}C^{-1}\right)\text{,}$$

where the material parameter μ is analogous to the shear modulus of linear elasticity, C is the right Cauchy–Green deformation tensor, I is the unity tensor, $I_{C}=\mathrm{tr}(C)$ and ${\mathrm{III}}_{C}=\det C$ are the first and third invariants of C, and *d* is the dimension of the domain (in our case d=2).

The domain was discretized using six different meshes of inf–sup stable $Q^{2}-Q^{1}$ Taylor–Hood quadrilateral elements [26]. For the weakly penalized formulation, a quadratic interpolation was used for the displacement field and a discontinuous linear interpolation was used for the pressure. The actual solution was approximated using the LM and PL solution (for the incompressible and nearly-incompressible comparison respectively) on a finer mesh (mesh_7_), with a cubic interpolation for the displacement and a quadratic interpolation for the pressure. Fig. 2(b) presents the number of elements in all seven meshes, and the corresponding degrees of freedom when the LM, PL, penalty and weakly penalized methods were used.

#### 3.1.2 Cardiac mechanics in the left ventricle

**Fig. 3:**
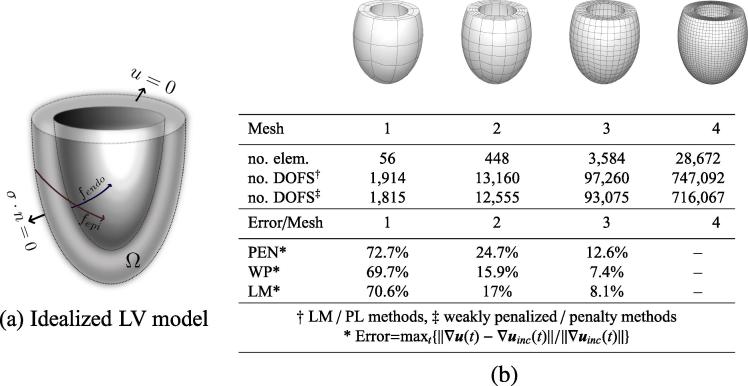
Discretization of the cardiac model: (*a*) The idealized LV was modeled as a thick-walled ellipsoid truncated at $\frac{3}{4}$ of the total height. Typical cardiac dimensions were used (semi-major axis=8cm, semi-minor axis=5.5cm, wall thickness= 0.5cm at the apex, 1cm at the base). The red and blue curves denote the epicardium ($f_{\mathrm{epi}}$) and endocardium ($f_{\mathrm{endo}}$) fiber directions [63,7], respectively. Zero traction condition was applied on the epicardial surface, and the base was held fixed. (*b*) Number of elements and degrees of freedom (DOFS) in each discretization and error of the three methods when used in the cardiac cycle test ($k=10^{7}$ for the penalty (PEN) and weakly penalized (WP) methods). (For interpretation of the references to colour in this figure caption, the reader is referred to the web version of this article.)

The three methods were tested on a model of the passive inflation of a left ventricle under diastolic loading conditions. The left ventricle (LV) was modeled as a thick-walled truncated ellipsoid (Fig. 3(a)). A standard generic heterogeneous fiber field was used to represent the structure of the tissue [7], where the fiber angle varied linearly between 60° and -60°, from endocardium to epicardium [63].

Several hyperelastic constitutive laws have been proposed to model the myocardial tissue. In this work, the myocardium was modeled using the transversely isotropic exponential law introduced by Guccione et al. [3], a model used frequently in the literature.^4^ This constitutive law is defined with respect to the fiber coordinate system where a coordinate system aligned with local tissue structure is defined everywhere in the material by orthogonal fiber $\overset{ˆ}{f}$, sheet $\overset{ˆ}{s}$, and sheet normal $\overset{ˆ}{n}$ unit vectors [5,11]. Letting,

(53) $$E_{\mathrm{vw}}=E:(\overset{ˆ}{v}⊗\overset{ˆ}{w})\text{,}\;\overset{ˆ}{v}\text{,}\overset{ˆ}{w}\in \{\overset{ˆ}{f}\text{,}\overset{ˆ}{s}\text{,}\overset{ˆ}{n}\}\text{,}$$

denote the components of strain aligned with the local tissue structure directions, then the fiber oriented Green strain is,

(54) $$E_{F}=Q^{T}\mathrm{EQ}=\;\left(\begin{array}{ccc} E_{\mathrm{ff}} & E_{\mathrm{fs}} & E_{\mathrm{fn}}\\ E_{\mathrm{sf}} & E_{\mathrm{ss}} & E_{\mathrm{sn}}\\ E_{\mathrm{nf}} & E_{\mathrm{ns}} & E_{\mathrm{nn}} \end{array}\right)\text{,}$$

with $Q=[\overset{ˆ}{f}\text{,}\overset{ˆ}{s}\text{,}\overset{ˆ}{n}]$. The strain energy and second Piola stress tensor may then be written as [3],

$$\begin{array}{cc} & \Psi (E)=\frac{C}{2}(e^{Q}-1)\text{,}\;Q=(A\circ E_{F}):E_{F}\text{,}\\ & S={\mathrm{Ce}}^{Q}Q(A\circ E_{F})Q^{T}\text{,} \end{array}$$

where A stores the material parameters governing the stress response to strain in fiber/sheet/normal directions, *i.e.*

(55) $$A=\left(\begin{array}{ccc} b_{f} & b_{\mathrm{fs}} & b_{\mathrm{fs}}\\ b_{\mathrm{fs}} & b_{t} & b_{t}\\ b_{\mathrm{fs}} & b_{t} & b_{t} \end{array}\right)\text{.}$$

The parameters used were $C=1760\mathrm{Pa}\text{,}b_{f}=18.5\text{,}b_{t}=3.58\text{,}b_{\mathrm{fs}}=1.63$ [25]. The endocardial surface of the ventricular model was passively loaded to 3kPa (22.5mmHg), to cover normal and pathological LV functions at end diastole. The LV was inflated using 150 equal load steps, by setting the boundary traction t equal to the product of the pressure and the deformed surface normal.

In order to simulate a cardiac cycle, the cardiac model was modified to include myocardial contraction through an active tension generation model [10]. Active tension generation was incorporated into the cardiac model by the addition of the active stress in the fiber direction of the stress tensor. The cardiac model was also coupled to a Windkessel model representing the systemic circulation using the parameters given by Korakianitis et al. [64]. The coupling was enabled through the use of a Lagrange Multiplier which enforces the same rate of change of LV volume in the two models [65].

The LV was discretized using four different meshes of hexahedral elements (Fig. 3(b)). On the first three discretizations, a quadratic interpolation was used for the displacement. The pressure field was interpolated using linear continuous (for the LM/PL methods) and discontinuous (for the weakly penalized formulation) Lagrange polynomials. In the cardiac tests the results on the first three meshes were compared with the LM and PL solution on mesh_4_ (for the incompressible and compressible comparison respectively), where a quadratic-linear interpolation scheme was employed.

### 3.2 Numerical solution

The solid mechanics tests presented in Section 3.1 were used to test the convergence behavior of the methods discussed. The convergence rate of each method was acquired by observing the change in the error between a high resolution benchmark solution and the approximate solution, with mesh refinement. As compressible methods may be selected as an approximation to incompressible behavior, we tested the convergence characteristics of the penalty and weakly penalized approaches to the incompressible LM solution (*i.e.* k=∞ in Eq. (11)). We also examined the ability of these approaches to model compressible behavior, comparing the results with a fine grid compressible solution(s) (PL solution(s)). The error tolerance for these tests was set to $1\times 10^{-9}$.

The problems under consideration were implemented in **CHeart** –a multi-physics software tool based on [66–68,55] and expanded by the **CHeart** team at KCL. All problems were solved on a Dell OPTIPLEX 990, quad-core (Intel^®^ Core™ i7–2600 CPU @ 3.40 GHz), on an 2.1 GHz AMD Opteron™ Interlagos 32 processor and on an SGI with 640 2.67 GHz processors (Intel^®^ Xeon^®^ CPU E7–8837).

### 3.3 Numerical results for the convergence rates

**Fig. 4:**
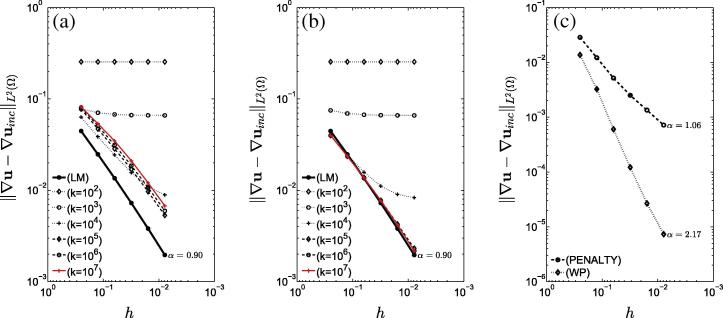
Comparison of the convergence behavior of the 3 methods on the two-dimensional elongation problem (the slope of these curves is denoted by α): The error between the (*a*) penalty and (*b*) weakly penalized approaches (u) and the incompressible fine grid solution ($u_{\mathrm{inc}}$) for six different values of the bulk modulus *k*. Convergence of the LM method is shown in black for comparison, whereas the red line represents the highest value of *k*. (*c*) Illustration of the error for penalty/weakly penalized ($k=10^{7}\;\text{Pa}$) approaches measured over a subset of the domain excluding the region around the four corners of the square.

**Fig. 5:**
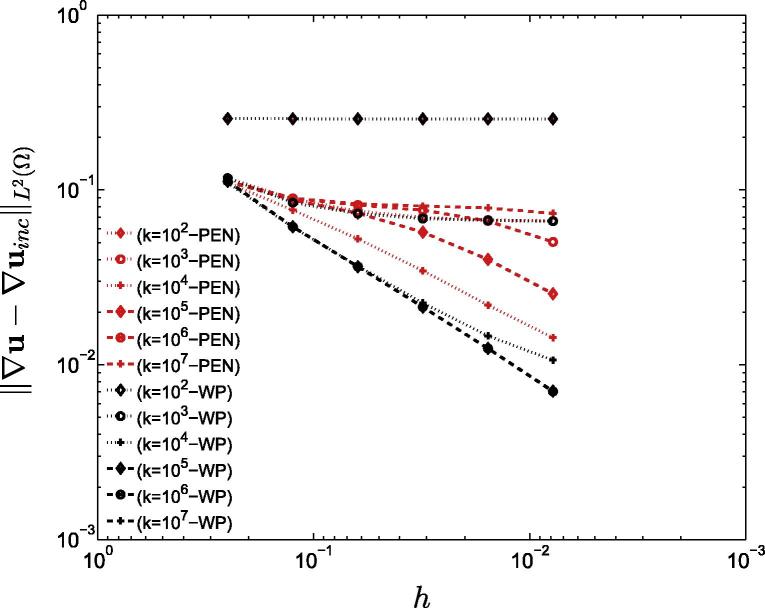
Comparison of the convergence behavior of the penalty and weakly penalized formulations when a lower order interpolation scheme is used: The errors between the (*red*) penalty and (*black*) weakly penalized forms (u) and the fine grid solution to the incompressible ($u_{\mathrm{inc}}$) elongation problem using linear interpolations are illustrated. (For interpretation of the references to colour in this figure caption, the reader is referred to the web version of this article.)

The LM, penalty and weakly penalized formulations were initially compared to the incompressible formulation of the elongation problem (Section 3.1.1). Fig. 4 compares the error of the penalty and weakly penalized methods in the solution of the incompressible elongation problem measured over the entire domain as well as a horizontal patch excluding the corners (where singularities in the solution occur). Finally, the importance of interpolation order is highlighted in Fig. 5, where linear interpolation was used for both penalty and weakly penalized formulations (where the local orthogonal projection was selected as the set of piecewise-discontinuous constants).

**Fig. 6:**
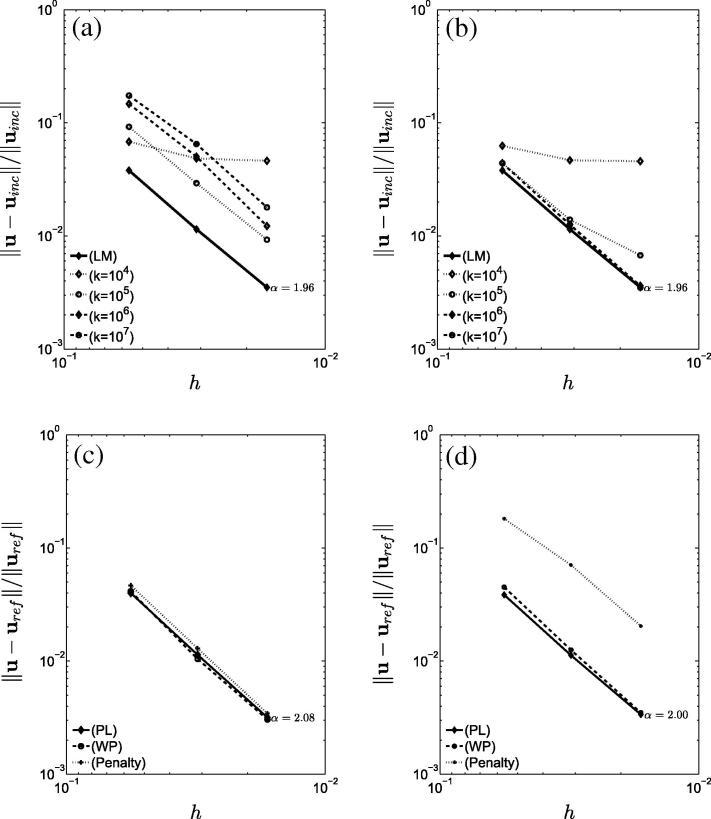
Comparison of the $L^{2}$ norm error of the various formulations compared to fine grid (*a*, *b*) incompressible and (*c*, *d*) compressible solutions of the passive inflation problem: (*Top*) Comparison as an approximation to the incompressible problem (LM solution) for (*a*) penalty and (*b*) weakly penalized methods for different *k* values. (*Bottom*) Comparison as an approximation to the compressible problem for PL, penalty and weakly penalized (WP) forms with (*c*) $k=10^{4}\;\text{Pa}$ and (*d*) $k=10^{7}\;\text{Pa}$. α denotes the slope of the LM and PL curves.

**Fig. 7:**
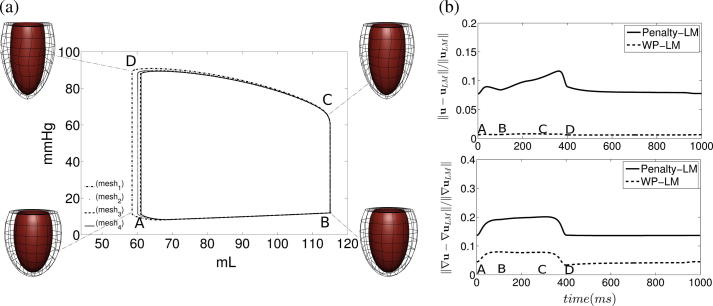
Comparison over the cardiac cycle: (*a*) Display of the pressure–volume loops of the weakly penalized solution of the cardiac cycle on the first three meshes. These pressure–volume loops are converging to the pressure–volume loop of the LM solution on mesh_4_. (*b*) The $L^{2}$ norm (*top*) and $H^{1}$ semi-norm (*bottom*) comparison of the displacement error between the penalty and weakly penalized formulations ($k=10^{7}$) in different phases of the cardiac cycle on mesh_2_. Letters A, B, C, D map the time in cycle (*b*) to cardiac phase on the pressure–volume loop (*a*).

Similar results can be observed in the passive inflation problem detailed in Section 3.1.2. Here convergence of the $L^{2}$-norm displacement error in the different methods is shown in Fig. 6 for both approximations to the fine grid incompressible (for $k\in [10^{4}\text{,}10^{7}]\;\text{Pa}$) and compressible passive inflation problems (with $k=10^{4}\;\text{Pa}$ and $k=10^{7}\;\text{Pa}$). The LM, penalty and weakly penalized methods were also compared on the cardiac cycle model showing consistent results to those illustrated in the passive inflation test. Representative results of this comparison are illustrated in Fig. 3(b), while Fig. 7(a) illustrates convergence of the weakly penalized pressure–volume loops with mesh refinement.

Examining the different effects of these methods on cardiac mechanics, we solved each model over a single cardiac cycle, comparing the differences in their behavior, using the LM method as the point of reference. The cardiac cycle was solved on an intermediate mesh (mesh_2_), using a quadratic-linear interpolation scheme for the displacement and pressure variables. Fig. 7 illustrates the pressure–volume loop derived from the coupled Windkessel-ventricle model as well as the differences between the LM model and both penalty and weakly penalized methods (with $k=10^{7}$) throughout the cycle. Fig. 9 shows the fiber strain, $E_{\mathrm{ff}}$ of the LM method, at the point where the most significant variations are observed (t=361ms after end diastole) as well as the absolute difference in fiber strain between the LM solution on every mesh and the respective penalty and weakly penalized approximations.

### 3.4 Numerical results for the efficiency of the different formulations

**Fig. 8:**
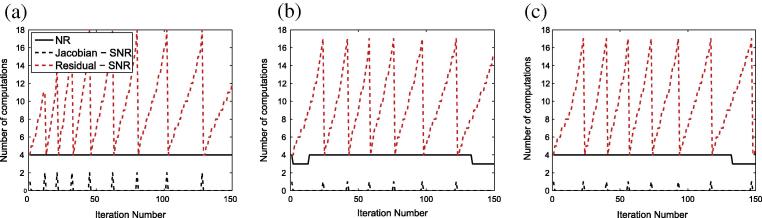
Comparison of the number of Jacobian and residual computations for (*a*) the penalty, (*b*) the weakly penalized and (*c*) the PL formulation. The methods are compared over the passive inflation simulation (Section 3.1.2) on mesh _2_ ($k=10^{7}$ for the penalty, weakly penalized and Perturbed Lagrangian (PL) methods). The solid black line presents the number of Jacobian and residual computations for the classic Newton–Raphson scheme and the dotted lines present the number of Jacobian (black line) and residual (red line) computations when the SNR scheme is applied. (For interpretation of the references to colour in this figure caption, the reader is referred to the web version of this article.)

The PL, penalty and weakly penalized formulations were compared in terms of their nonlinear convergence behavior. Representative values for the number of iterations of the Newton–Rapson scheme, the number of Jacobian matrix computations (and their respective times) along with the linear solution time and total time are presented in Table 1. The improvement in the efficiency of the different methods when the SNR scheme is applied is presented as well. Finally, the effect of the modifications we introduced in the SNR scheme for the penalty method can be deduced as the table compares the application of the SNR scheme to the penalty method with and without the introduced modifications. Note that although not presented here, the nonlinear behavior of the weakly penalized system when the SNR is applied without the introduced modifications is similar to that of the penalty method without the introduced modifications (PEN). Similar observations can be made using Fig. 8 which compares the number of Jacobian and residual computations when the classic Newton–Raphson and the SNR scheme are used for the different methods.

## 4 Discussion

### 4.1 Comparison of the methods for modeling incompressibility

Examining the ability of all approaches to model imcompressibility, as we noted in the introduction the most straightforward is the incompressible LM method which enforces weak incompressibility. However, we could consider the compressible penalty and weakly penalized approaches as approximations to the incompressible system. With this in mind, from Eq. (11), the error for any method should converge to zero with a rate proportional to 1/k. For the penalty method, we see from Fig. 4(a) and Fig. 6(a) that the error relative to the incompressible solution was generally higher and increased with increasing *k*, as a result of the well-known locking phenomena associated with displacement-only formulations. In the cardiac model, with $k=10^{7}$ the error was actually uniformly worse than all other values of the parameter at almost all levels of refinement, making the selection of an appropriate *k* to model incompressible behavior non-trivial.

As discussed in Section 2.2, we initially hypothesized that issues affiliated with the penalty method could be circumvented by projecting the constraint using an orthogonal projection operator, $\pi_{W_{\mathrm{loc}}}$, resulting in the displacement-based formulation suggested by Bercovier [50] and others [48,49]. Indeed, from Fig. 4(b) and Fig. 6(b), we see that as *k* increased, the error in the approximation decreased proportionally to 1/k and became indistinguishable from the convergence of the LM method itself. The existence of a *k*-dependent error bound for the weakly penalized approach enables regulation of the error by an appropriate choice of *k*. Moreover, due to its dependence on the discretization, the error showed plateauing behavior for values of *k* which incured an error larger than the error associated with the discretization.

The locking behavior of the penalty method is observed to worsen with lower order as shown in Fig. 5, where linear elements were used. In this case, as the bulk modulus increased, the rate of convergence observed in the penalty method deteriorated to nearly zero. In contrast, the weakly penalized approach exhibited consistent linear convergence for $k>10^{5}$.

We notice that for both elongation/cardiac problems, the rates of convergence from all methods were not optimal as we would expect based on the error estimates [61]. As the sub-optimal convergence rates appear in the application of all methods, we can assume that this is not a method-dependent issue. We believe that it is due to singularities in the two problems which limit convergence. In the elongation problem, singularities occur at all corners of the domain. Measuring convergence in a horizontal patch excluding corners as shown in Fig. 4(c), we see that for $k=10^{7}$ the rate of convergence in the weakly penalized method is restored to the expected order $O(h^{2})$ (for the $H^{1}$ semi-norm). In contrast, due to locking, no improvement to the rate of convergence is observed in the penalty approach. In the cardiac model, sub-optimal convergence is due to fixing the base plane of the model and the singularity in the fiber field near the apex. Even though the specific boundary condition and fiber field incur singularities, they were chosen because of their frequent use in cardiac models.

### 4.2 Comparison of the methods for modeling compressibility

Similar conclusions can be deduced by the application of the PL, penalty and weakly penalized formulations in the solution of compressible problems. In compressible problems, the three formulations should provide consistent results for low and moderate values of *k*. This is observed in Fig. 6(c). While we observed flattening of the convergence behavior to the incompressible solution for $k=10^{4}$ in both penalty and weakly penalized methods (and, though not shown, for PL), we see uniform and consistent convergence to the compressible solution.

Increasing *k*, however, caused deterioration in the convergence of the penalty method as shown in Fig. 6(d). Here, the error increased by almost an order of magnitude, while convergence remains consistent between the PL and weakly penalized methods. We note that identical behavior was observed in the 2D elongation problem and, while measurement of the error excluding corners, restored optimal convergence rates in the PL and weakly penalized methods, no improvement in the rate was observed in the penalty method.

### 4.3 Comparison over the cardiac cycle

**Fig. 9:**
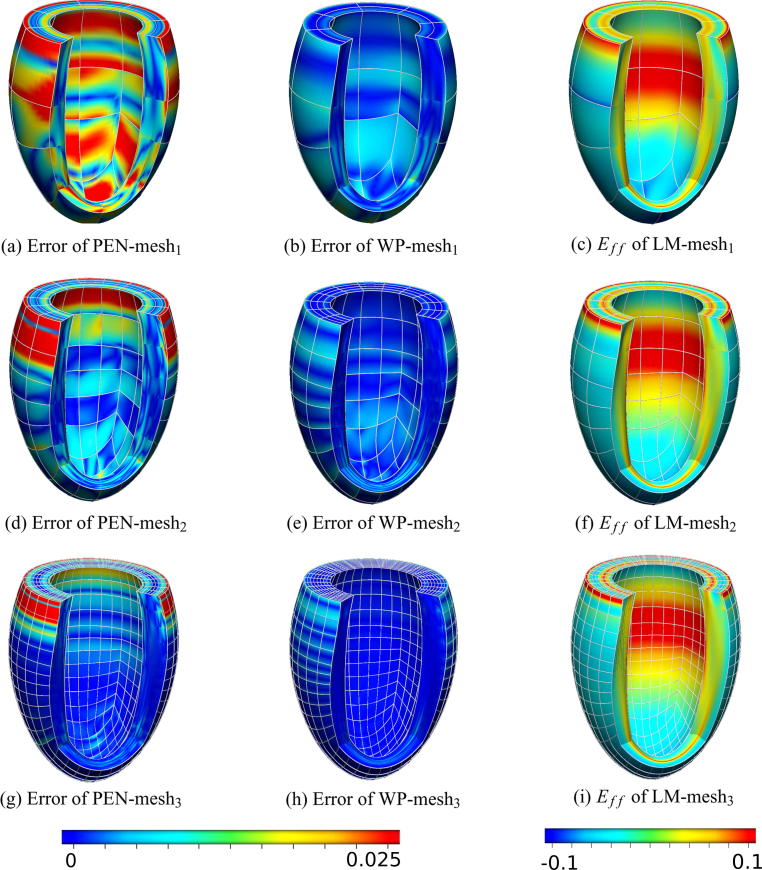
Illustration of the absolute difference (error) in fiber strain ($E_{\mathrm{ff}}$) at 361ms, between the Penalty and LM method (*a, d, g*) and the weakly penalized and LM method (*b, e, h*) on the first three meshes (colors representing values of the error between 0 (blue) and 0.025 (red)). Additionally, the fiber strain for the LM method on each mesh is displayed (*c, f, i*), with colors ranging from blue (-0.1) to red (0.1). Figures created in CMGUI [69]. (For interpretation of the references to colour in this figure caption, the reader is referred to the web version of this article.)

In Fig. 7(b), we compare all methods over a cardiac cycle plotting the difference between both penalty and weakly penalized approaches (with $k=10^{7}$) and the incompressible LM method on the same discretization. This comparison was performed on an intermediate mesh, mesh_2_, consisting of 448 elements. Here we see that the LM and penalty methods differ in the $H^{1}$ semi-norm (which is indicative of errors we could expect in strain) by up to 20%, while the peak difference between weakly penalized and LM approaches remains below 8%. These differences in strain occured primarily during the systolic phase, with decreased error through the rest of the cardiac cycle. Similar conclusions can be deduced from the bulk behavior of these models as seen in Fig. 9, presenting a larger difference in fiber strain between the penalty and LM methods than the weakly penalized and LM methods. Interestingly, in this case the error of both the penalty and weakly penalized formulations decreased with mesh refinement.

The influence of these effects is heavily dependent on the *k* chosen for the model. Considering convergence (*i.e.* mesh_3_ with fine grid mesh_4_) of the compressible model over the cardiac cycle (even though not shown here), the maximum error for $k=10^{7}$ was ∼1% for the weakly penalized and LM methods and ∼10% for the penalty method. However, for $k=10^{5}$ the error for weakly penalized and LM methods remained around ∼1% while the error observed in the penalty method dropped to ∼3%. As the bulk modulus represents the tissue’s resistance to compression, its value is tied to the other cardiac constitutive parameters. Thus the influence of locking in the penalty method depends on the level of compressibility which is acceptable in the model. In general it seems that as $k/C>10^{3}$, where *C* is the bulk scaling on the strain energy in Section 3.1.2, locking becomes increasingly more predominant.

### 4.4 Comparison of linearized systems and solution

In Section 2.5.2, we outlined the solution procedure for the weakly penalized formulation illustrating that the linearized system involves only the body displacement, $u^{h}$. Considering the Jacobian for the LM ($J_{\lambda }$), and penalty methods ($J_{k}$), shown in Eq. (56), the LM formulation has an indefinite saddle point structure while the penalty method adds to the principle A- block,

(56) Jλ=ABB^0,Jk=A+P.

Similar to $J_{k}$, the Jacobian of the weakly penalized formulation shown in Eq. (34) also augments the A- block with a matrix, C which, by construction, is symmetric positive semi-definite. Further, the Jacobian of the PL method augments the zero block matrix with a *k*-dependent term, avoiding the indefinite nature of the LM Jacobian (the Jacobians of the different formulations are outlined in Appendix A).

The structure of these systems has a significant impact on their solution. While the actual system sizes (shown in Fig. 2(b) and 3(b)) are not substantially different, the indefinite structure of $J_{\lambda }$ makes it more challenging to solve, requiring direct methods, “sophisticated” preconditioners or splitting schemes [70]. In contrast, the penalty, PL and weakly penalized strategies are more straightforward in structure, making them more ammenable to classic preconditioning strategies. However, as the bulk modulus *k* increases, care must be taken to deal with the conditioning of the linear system.

In addition to having contrasting linear structure, the methods also exhibited differing convergence behavior in the Newton–Raphson scheme outlined in Section 2.5.1.^5^ In general, the non-linear convergence of the weakly penalized formulation averaged ∼3.81 iterations per load step when the classic Newton–Raphson scheme was employed (Table 1). The modifications we introduced in the weakly penalized form (Section 2.5.2), enhanced the numerical ability of the scheme, which exhibited marginally better non-linear convergence behavior than that of the PL method.

Furthermore, the PL and weakly penalized forms were able to exploit the Jacobian re-use strategy (Shamanskii–Newton–Raphson scheme), leading to approximately 93% decrease in the computational time of the Jacobian matrix J (build and solution) and a 94% reduction in the total time per loading step (for the weakly penalized form). By modifying the weakly penalized scheme to avoid the high sensitivity to the bulk modulus associated with displacement formulations, the weakly penalized formulation allows efficient re-use of the Jacobian matrix, whereas the performance of the penalty formulation (PEN) is not significantly improved when the SNR scheme is applied. When these modifications were also extended to the penalty method (PEN-MOD), they resulted in significant improvements in both the computational time of the Jacobian matrix J (87% decrease) and the total time per loading step (89% decrease) compared to the classic Newton–Raphson scheme.

Similar conclusions can be deduced from Fig. 8 which compares the Jacobian and residual computations over the iteration number, with and without the SNR scheme. Clearly, the SNR scheme significantly reduces the number of Jacobian computations for all methods. It is important to note that these observations are consistent in all formulations, indicating that the modifications we introduced in the SNR scheme for both the weakly penalized and penalty formulations were able to significantly improve the performance of the methods.

### 4.5 Weakly penalized formulation

In Section 2.1.1 we illustrated how the energy functional for a hyperelastic solid can be written consistently for both penalty and LM methods by choosing both an appropriate space of solutions, X, and orthogonal projection, $\pi_{W}$. In the finite element context, we showed that in the LM method both X and $\pi_{W}$ were necessarily discretized as both the displacement and pressure variables need to be computed, while in the penalty formulation the orthogonal projection is not necessarily discretized as the only unknown variable is the displacement (Section 2.2). As we have shown, this discretization of the orthogonal projection can restrict the approximation space $X^{h}$ for high values of *k*.

To circumvent this issue while retaining the single field approach, in Section 2.3 we apply a displacement-only formulation introduced by Bercovier [50] and others [48,49] which uses a localized discrete orthogonal projection operator, $\pi_{W_{\mathrm{loc}}}$. Similar to augmented Lagrangian and reduced integration techniques [26], the aim of the discrete projection is to weaken the compressible/incompressible constraint, thereby enhancing the approximation space in the limit as *k* gets large. Furthermore, by appropriate restructuring of the weakly penalized system, we avoid the poor nonlinear convergence for high bulk modulus associated with displacement-only formulations. As shown in Figs. 4 and 6, the weakly penalized formulation restores convergence behavior while maintaining the simplicity of a single field approach. Finally, viewing the various methods under the same generalized framework allows us to extend the SNR modifications of the weakly penalized form to the penalty approach, significantly improving the computational performance of the scheme.

A convenient feature of the weakly penalized approach is that it enables more straightforward analysis by tapping into known finite element spaces. Though for uniqueness inf–sup stability is not necessary, this condition ensures optimal convergence in the null space of $\pi_{W}$ for linear problems, for appropriately chosen spaces. In the examples presented here, the projection was chosen to be one polynomial order less and piece-wise discontinuous. Though this pairing is not inf–sup stable, for the quadrilateral and hexahedral elements considered, this restored convergence. Another convenient choice are Nicolaides–Boland [60] elements, which give consistent results to those presented here.

## 5 Conclusion

In this paper we compared the use of different methods for approximating incompressible and compressible tissue mechanics in the heart. Noting that the choice of model is governed by both model validity and numerical considerations, we assessed the use of Lagrange (LM and PL) and penalty methods as models of both incompressible and compressible behavior. Motivated by the classic locking phenomena observed for linear mechanics [26,27], we apply an enhancement of the Bercovier [50] formulation which enables the single field approach while providing similar convergence behavior to the LM method. To the best of our knowledge this is the first application of this approach on heart models.

Observing the convergence behavior of these methods on a simple solid mechanics test and on cardiac models, we highlight the fact that the LM and penalty methods, although often used equivalently in cardiac mechanics, may present significant variations in results. This is due to the well-known deterioration of the convergence behavior of the penalty method for large values of the bulk modulus. Indeed in both the 2D elongation problem and the cardiac models, the penalty method generally has a larger error than the other two methods for all values of the bulk modulus. In contrast, the single field weakly penalized approach provides both improved rates of convergence and avoids issues associated with locking phenomena over these test problems. Further modifications introduced in this work enhance the computational performance of the numerical scheme, by allowing efficient application of the SNR re-use strategy and significantly reducing the computational time. The weakly penalized formulation can therefore provide an accurate and computationally inexpensive method that can be used to deal with incompressibility and near incompressibility in problems of cardiac mechanics and solid mechanics in general.

## Figures and Tables

**Table 1:** Comparison of average number of Newton–Raphson iterations and Jacobian computations per load step as well as their respective average times between Newton–Raphson and Shamanskii–Newton–Raphson schemes. The efficiency of the SNR scheme with (PEN-MOD) and without (PEN) the introduced modifications on the penalty method is presented as well. The total solve time per load step and the total time per load step are illustrated as well. This comparison was performed on the passive inflation test (Section 3.1.2) on mesh_2_ ($k=10^{7}$ for the penalty, weakly penalized and Perturbed Lagrangian (PL) methods), the simulations were run on a single processor and a direct solver was used.

	J compute time [s]^a^	J computations^a^	R compute time [s]^a^	R Computations a	Solve time [s]^a^	Total time [s]^a^
*Newton–Raphson*						
PEN	181.55	4	1.93	4	47.51	231.13
WP	242.05	3.81	2.42	3.81	41.46	286.06
PL	246.77	3.88	2.49	3.88	45.36	294.75

*Shamanskii–Newton–Raphson*						
PEN	25.80	0.46	11.67	10.82	7.2	44.81
PEN-MOD	10.14	0.113	14.07	9.51	1.76	26.08
WP-MOD	3.95	0.047	13.19	9.79	1.18	18.47
PL	4.66	0.053	13.43	9.88	1.37	19.61

a Times/iterations given as the average per load step.

## Appendix A Discrete weak forms/Jacobian matrices for the penalty, PL and LM methods

The aim of this section is to provide the discrete weak forms of the formulations implemented and used in this work. A detailed derivation of the weak forms of the penalty, LM and PL methods is provided in various textbooks [45,29]. Specifically, the weak form for the penalty method is acquired by requiring that the directional derivative of the penalty functional $\Pi_{k}$ vanishes for all arbitrary directions $\delta u^{h}$ in the homogeneous zero Dirichlet space $X_{0}^{h}$, *i.e.*

(A.1) $$D\Pi_{k}(u^{h})[\delta u^{h}]=0\text{,}\;\forall \delta u^{h}\in X_{0}^{h}\text{.}$$

Similarly, the PL method requires that,

(A.2) $$D\Pi_{\lambda }(u^{h}\text{,}\lambda^{h})[\delta u^{h}\text{,}\delta q^{h}]=0$$

for every $(\delta u^{h}\text{,}\delta q^{h})\in X_{0}^{h}\times W^{h}$. The discrete weak forms for the penalty, PL and LM systems can also be written in operator notation as,

(A.3) $$A(u^{h}\text{,}\delta u^{h})+P(u^{h}\text{,}\delta u^{h})=F(\delta u^{h})\text{,}$$

(A.4) A(uh,δuh)+B(ph,uh,δuh)+B^(uh,δph)-1kM(ph,δph)=F(δuh),

(A.5) A(uh,δuh)+B(ph,uh,δuh)+B^(uh,δph)=F(δuh),

where the operators A,P,F,B,B^,M, and *C* are defined as,

A(uh,δuh)=∫ΩFhSh:∇δuhdV,P(uh,δuh)=∫ΩkJuh(Juh-1)Fh-T:∇δuhdV,F(δuh)=∫Ωf·δuhdV+∫∂Ωt·δuhdA,B(λh,uh,δuh)=∫ΩλhJuhFh-T:∇δuhdV,B^(uh,δqh)=∫Ωδqh(Juh-1)dV,M(λh,δqh)=∫ΩλhδqhdV,

where $F_{h}=\nabla u^{h}+I$ and $S_{h}$ represent the discrete deformation gradient and second Piola stress tensors, respectively.

The Jacobian of these formulations is then derived by taking the directional derivative of the discrete weak forms with respect to displacement (and pressure). For the penalty method, the Jacobian can not be derived analytically due to the nonlinearity of hyperelastic laws and is usually estimated by the finite difference approximation of the gradient of the operators A,P and *F*:

(A.6) $$J_{k}=A+P\text{,}$$

$${\left[A\right]}_{\mathrm{ij}}=\frac{1}{∊}\left(A(u^{h}+∊\phi_{u}^{j}\text{,}\phi_{i})-F(u^{h}+∊\phi_{u}^{j}\text{,}\phi_{i})-A(u^{h}-∊\phi_{u}^{j}\text{,}\phi_{i})+F(u^{h}-∊\phi_{u}^{j}\text{,}\phi_{i})\right)\text{,}$$

(A.7) $${\left[P\right]}_{\mathrm{ij}}=\frac{1}{∊}(P(u^{h}+∊\phi_{u}^{j}\text{,}\phi_{i})-P(u^{h}-∊\phi_{u}^{j}\text{,}\phi_{i}))\text{,}$$

where we note that, the operator *F* is included in the Jacobian estimation as it often depends on u.

The Jacobian of the PL method is written in matrix form as

(A.8) JPL=ABB^-1kM,

where the block matrices A,B and B^ are defined as

(A.9) $$\begin{array}{cc} & {\left[A\right]}_{\mathrm{ij}}=\frac{1}{∊}\left(A(u^{h}+∊\phi_{u}^{j}\text{,}\phi_{i})+B(\lambda^{h}\text{,}u^{h}+∊\phi_{u}^{j}\text{,}\phi_{i})-F(u^{h}+∊\phi_{u}^{j}\text{,}\phi_{i})-A(u^{h}-∊\phi_{u}^{j}\text{,}\phi_{i})-B(\lambda^{h}\text{,}u^{h}-∊\phi_{u}^{j}\text{,}\phi_{i})+F(u^{h}-∊\phi_{u}^{j}\text{,}\phi_{i})\right)\text{,}\\ & {\left[B\right]}_{\mathrm{ij}}=B(\phi_{w}^{j}\text{,}u^{h}\text{,}\phi_{u}^{i})\text{,} \end{array}$$

(A.10) [B^]ij=B(ϕwi,uh,ϕuj),

The block matrix M is the mass matrix defined in Eq. (20).

The Jacobian matrix of the LM formulation can be derived from the Jacobian of the PL formulation by assuming k→∞, as

(A.11) Jλ=ABB^0,

where the 0 block matrix results in a non-positive definite Jacobian matrix which may cause numerical difficulties.

## Appendix B Error estimate for the generalized weakly penalized form

In this section we present an error estimate for the generalized weakly penalized form and illustrate how this may be used to derive estimates for the Perturbed Lagrangian, incompressible and/or nearly incompressible forms. For ease, a linear elastic material is considered with Homogenous Dirichlet boundary conditions, giving the continuous weakly penalized problem: find $u_{k}\in X_{0}=\{u\in {\left[H^{1}(\Omega )\right]}^{d}|u=0\;\mathrm{on}\;\Gamma \}$ such that,

(B.1) $$\int_{\Omega }{\mathrm{EDu}}_{k}:\mathrm{Dw}\mathrm{dV}+\int_{\Omega }k\pi (\nabla \cdot u_{k})\nabla \cdot w\mathrm{dV}=\int_{\Omega }f\cdot w\mathrm{dV}\text{,}\;\forall w\in X_{0}\text{,}$$

where $E:\Omega \to R^{d\times d}$ is the elasticity tensor, D(·)=1/2(∇(·)+(·)∇),k>0 is the bulk modulus of the material, π the projection operator (in this case the $L^{2}(\Omega )$ projection) and $f\in L^{2}(\Omega )$ is given data. Here we assume E is a symmetric positive definite matrix satisfying, for any $v\text{,}w\in X_{0}$, the linear coercivity and continuity conditions (where $\|\cdot {\|}_{1}$ is the norm on $H^{1}(\Omega )$),

(B.2) $$\alpha \|v{\|}_{1}^{2}⩽\int_{\Omega }\mathrm{EDv}:\mathrm{Dv}\mathrm{dV}\text{,}\;\int_{\Omega }\mathrm{EDv}:\mathrm{Dw}\mathrm{dV}⩽\gamma \|v{\|}_{1}\|w{\|}_{1}\text{.}$$

We note that here, given $u\in X_{0}(\Omega )$, using Korn’s inequality [71] there exists an equivalence between ‖Dv‖ and $\|v{\|}_{1}$ (which, for ease, are included in these bounds). Due to the coercivity/continuity conditions, the existence and uniqueness of solutions is ensured by Lax–Milgram lemma [61].

Noting that the space $X_{0}=X_{0\text{,}\mathrm{div}}⊕{\left(X_{0\text{,}\mathrm{div}}\right)}^{⊥}$ (with $X_{0\text{,}\mathrm{div}}=H_{0\text{,}\mathrm{div}}^{1}(\Omega )$) is separable into divergence free and non-divergence free functions, we may also write $u_{k}=u_{k}^{*}+(1/k)u_{k}^{⊥}$ with $u_{k}^{*}\in X_{0\text{,}\mathrm{div}}$ and $u_{k}^{⊥}\in {\left(X_{0\text{,}\mathrm{div}}\right)}^{⊥}$, which satisfies,

(B.3) $$\int_{\Omega }\mathrm{ED}\left(u_{k}^{*}+\frac{1}{k}u_{k}^{⊥}\right):\mathrm{Dw}\mathrm{dV}+\int_{\Omega }\pi (\nabla \cdot u_{k}^{⊥})\nabla \cdot w\mathrm{dV}=\int_{\Omega }f\cdot w\mathrm{dV}\text{,}\;\forall w\in X_{0}\text{.}$$

The weakly penalized form introduced in Eq. (B.1) can be easily related to the equivalent continuous Perturbed Lagrangian, Penalty and Lagrange Multiplier forms (with appropriate selection of *k*). As any $w\in X_{0}$ satisfies $\nabla \cdot w\in L^{2}(\Omega )$, it is clear that $\pi (\nabla \cdot u_{k})=\nabla \cdot u_{k}$. Inserting this relation into Eq. (B.1) yields the continuous penalty form. Moreover, from the continuous constraint of the Perturbed Lagrangian we observe that $\pi (\nabla \cdot u_{k})=\lambda /k$ (or $\pi (\nabla \cdot u_{k}^{⊥})=\lambda$), enabling condensation of the PL form into the weakly penalized variant. Finally, in the limit as k→∞ it is clear that in Eq. (B.1) $u\in X_{0\text{,}\mathrm{div}}$, yielding equivalence with the LM form [50].

In the following, we shall consider an error estimate for the discrete weakly penalized problem: find $u^{h}\in X_{0}^{h}\subset X_{0}$ such that,

(B.4) $$\int_{\Omega }{\mathrm{EDu}}^{h}:{\mathrm{Dw}}^{h}\mathrm{dV}+\int_{\Omega }k_{h}\pi^{h}(\nabla \cdot u^{h})\nabla \cdot w^{h}\mathrm{dV}=\int_{\Omega }f\cdot w^{h}\mathrm{dV}\text{,}\;\forall w^{h}\in X_{0}^{h}\text{,}$$

where E is the elasticity tensor and initial data f are defined as above. Here we let $k_{h}>0$ denote the bulk modulus of the discrete model, acknowledging that we may choose (for numerical convenience, for example) to compute solutions using a bulk modulus which is different from the continuous model (*i.e.* $k_{h}\;\neq \;k$). Further, we consider

(B.5) $$\pi^{h}:L^{2}(\Omega )\to S\subseteq L^{2}(\Omega )\text{,}$$

to be the $L^{2}$ projection operator onto the subspace $S\subseteq L^{2}(\Omega )$. As a consequence, it is clear that,

(B.6) $$\|\pi^{h}(g)\|⩽\|g\|\text{,}\;g\in L^{2}(\Omega )\text{,}$$

(where ‖·‖ denotes the $L^{2}(\Omega )$ norm). Moreover, due to symmetry in the discrete projection, for any $v^{h}\text{,}w^{h}\in X_{0}^{h}$,

(B.7) $$\int_{\Omega }\pi^{h}(\nabla \cdot v^{h})\nabla \cdot w^{h}\mathrm{dV}=\int_{\Omega }\pi^{h}(\nabla \cdot v^{h})\pi^{h}(\nabla \cdot w^{h})\mathrm{dV}\text{.}$$

From Eqs. (B.2), (B.6) and (B.7), the discrete form retains continuity and coercivity yielding a unique solution as in the continuous case. Similar to above, equivalence of this form to other methods (in their discrete form) can be obtained with appropriate selections of $\left(S\text{,}k_{h}\right)$, *i.e.* LM $\left(W^{h}\text{,}\infty \right)$, PL $\left(W^{h}\text{,}k\right)$, and Penalty $\left(L^{2}(\Omega )\text{,}k\right)$.

Finally, we may derive the following error estimate between the weakly penalized form in Eq. (B.4) and the continuous form introduced in Eq. (B.1). Lemma 1 *Let* $u_{k}$ *and* $u^{h}$ *be the solutions to Eqs.* (B.1) and (B.4)*, with* $u_{k}=u_{k}^{*}+(1/k)u_{k}^{⊥}$ *with* $u_{k}^{*}\in X_{0\text{,}\mathrm{div}}$ *and* $u_{k}^{⊥}\in {\left(X_{0\text{,}\mathrm{div}}\right)}^{⊥}$*. Then the difference between discrete and continuous solutions (when* $k=k_{h}$*) is given by,*

(B.8) ‖ukh-uh‖1⩽Cinfyh∈X0,divhzh∈X0,divh⊥‖πh(∇·ukh⊥)-∇·ukh⊥‖+‖ukh∗-yh‖1+1kh+1‖ukh⊥-zh‖1,

*where* $X_{0}^{h}=X_{0\text{,}\mathrm{div}}^{h}⊕{\left(X_{0\text{,}\mathrm{div}}^{h}\right)}^{⊥}$ *and* $X_{0\text{,}\mathrm{div}}^{h}≔\{v^{h}\in X_{0}^{h}|\;\|\pi^{h}(\nabla \cdot v^{h})\|=0\}$ *are the discrete projected null and perpendicular spaces, respectively. When* $k\;\neq \;k_{h}$*,*

(B.9) $$\|u_{k}-u^{h}{\|}_{1}⩽\|u_{k_{h}}-u^{h}{\|}_{1}+C_{0}\max\left\{\frac{1}{k}\text{,}\frac{1}{k_{h}}\right\}\text{,}$$

*with* $C\text{,}C_{0}>0$ *being positive constants independent of h.* Proof Selecting $w=w^{h}$ in Eq. (B.1) and noting equality of the RHS with Eq. (B.4), it is clear that,

(B.10) $$\int_{\Omega }{\mathrm{EDu}}^{h}:{\mathrm{Dw}}^{h}\mathrm{dV}+\int_{\Omega }k_{h}\pi^{h}(\nabla \cdot u^{h})\nabla \cdot w^{h}\mathrm{dV}=\int_{\Omega }{\mathrm{EDu}}_{k_{h}}:{\mathrm{Dw}}^{h}\mathrm{dV}+\int_{\Omega }k_{h}\pi (\nabla \cdot u_{k_{h}})\nabla \cdot w^{h}\mathrm{dV}\text{.}$$

Letting $\varepsilon_{h}=u^{h}-v^{h}$ and $e=u_{k_{h}}-v^{h}$ for some $v^{h}\in X_{0}^{h}$, we may express Eq. (B.10) as,

(B.11) $$\int_{\Omega }\mathrm{ED}\varepsilon_{h}:{\mathrm{Dw}}^{h}\mathrm{dV}+\int_{\Omega }k_{h}\pi^{h}(\nabla \cdot \varepsilon_{h})\nabla \cdot w^{h}\mathrm{dV}=\int_{\Omega }\mathrm{EDe}:{\mathrm{Dw}}^{h}\mathrm{dV}+\int_{\Omega }k_{h}\pi^{h}(\nabla \cdot e)\nabla \cdot w^{h}\mathrm{dV}+\int_{\Omega }k_{h}[\pi (\nabla \cdot u_{k_{h}})-\pi^{h}(\nabla \cdot u_{k_{h}})]\nabla \cdot w^{h}\mathrm{dV}\text{.}$$

Selecting $w^{h}=\varepsilon_{h}$, applying the coercivity/continuity conditions in Eq. (B.2), Cauchy Schwarz Inequality and noting the symmetry condition in Eqs. (B.7) and (B.11), (B.11) may be further reduced,

(B.12) $$\alpha \|\varepsilon_{h}{\|}_{1}^{2}+k_{h}\|\pi^{h}(\nabla \cdot \varepsilon_{h}){\|}^{2}⩽\int_{\Omega }\mathrm{EDe}:D\varepsilon_{h}\mathrm{dV}+\int_{\Omega }k_{h}\pi^{h}(\nabla \cdot e)\nabla \cdot \varepsilon_{h}\mathrm{dV}+\int_{\Omega }k_{h}[\pi (\nabla \cdot u_{k_{h}})-\pi^{h}(\nabla \cdot u_{k_{h}})]\nabla \cdot \varepsilon_{h}\mathrm{dV}⩽\gamma \|e{\|}_{1}\|\varepsilon_{h}{\|}_{1}+k_{h}\left[\left\|\pi^{h}(\nabla \cdot e)\|+\|\pi (\nabla \cdot u_{k_{h}})-\pi^{h}(\nabla \cdot u_{k_{h}})\right\|\right]\|\nabla \cdot \varepsilon_{h}\|\text{.}$$

Noting $\|\nabla \cdot w\|⩽\sqrt{d}\|w{\|}_{1}$ for any $w\in X_{0}$, Eq. (B.12) yields,

(B.13) $$\alpha \|\varepsilon_{h}{\|}_{1}⩽\gamma \|e{\|}_{1}+\sqrt{d}k_{h}\left[\left\|\pi^{h}(\nabla \cdot e)\|+\|\pi (\nabla \cdot u_{k_{h}})-\pi^{h}(\nabla \cdot u_{k_{h}})\right\|\right]\text{.}$$

Further, observing that $\varepsilon_{h}=u^{h}-u_{k_{h}}+e$ and applying the reverse triangle inequality,

(B.14) $$\alpha \|u_{k_{h}}-u^{h}{\|}_{1}⩽(\gamma +\alpha )\|e{\|}_{1}+\sqrt{d}k_{h}\left[\left\|\pi^{h}(\nabla \cdot e)\|+\|\pi (\nabla \cdot u_{k_{h}})-\pi^{h}(\nabla \cdot u_{k_{h}})\right\|\right]\text{.}$$

Finally, splitting $v^{h}≔y^{h}+(1/k_{h})z^{h}$ where $(y^{h}\text{,}z^{h})\in X_{0\text{,}\mathrm{div}}^{h}\times {\left(X_{0\text{,}\mathrm{div}}^{h}\right)}^{⊥}$ and $u_{k_{h}}=u_{k_{h}}^{*}+(1/k_{h})u_{k_{h}}^{⊥}$ as in Eq. (B.3) and applying the triangle inequality,

(B.15) $$\|u_{k_{h}}-u^{h}{\|}_{1}⩽\frac{\left(\gamma +\alpha \right)}{\alpha }\|e{\|}_{1}+\frac{\sqrt{d}k_{h}}{\alpha }\left[\left\|\pi^{h}(\nabla \cdot e)\|+\|\pi (\nabla \cdot u_{k_{h}})-\pi^{h}(\nabla \cdot u_{k_{h}})\right\|\right].⩽\frac{\left(\gamma +\alpha \right)}{\alpha }\left(\|u_{k_{h}}^{*}-y^{h}{\|}_{1}+\frac{1}{k_{h}}\|u_{k_{h}}^{⊥}-z^{h}{\|}_{1}\right)+\frac{\sqrt{d}}{\alpha }\left[\left\|\pi^{h}(\nabla \cdot [u_{k_{h}}^{⊥}-z^{h}])\|+\|\pi (\nabla \cdot u_{k_{h}}^{⊥})-\pi^{h}(\nabla \cdot u_{k_{h}}^{⊥})\right\|\right].⩽C\left(\left\|u_{k_{h}}^{*}-y^{h}{\|}_{1}+\left(\frac{1}{k_{h}}+1\right)\|u_{k_{h}}^{⊥}-z^{h}{\|}_{1}+\|\nabla \cdot u_{k_{h}}^{⊥}-\pi^{h}(\nabla \cdot u_{k_{h}}^{⊥})\right\|\right)\text{.}$$

where C=max{d/α,(γ+α)/α}. Taking the infimum, we arrive at the first result of Lemma 1. The second result follows directly from [50] where the error between $u_{\infty }$ and $u_{k}$ satisfies the estimate,

$$\|u_{\infty }-u_{k}{\|}_{1}⩽C/k$$

for any k>0. Consequently,

(B.16) $$C/k⩾\|u_{\infty }-u_{k}{\|}_{1}=\|u_{\infty }-u_{k_{h}}+u_{k_{h}}-u_{k}{\|}_{1}⩾\|u_{k_{h}}-u_{k}{\|}_{1}-\|u_{\infty }-u_{k_{h}}{\|}_{1}=\|u_{k_{h}}-u^{h}+u^{h}-u_{k}{\|}_{1}-\|u_{\infty }-u_{k_{h}}{\|}_{1}⩾\|u^{h}-u_{k}{\|}_{1}-\|u_{k_{h}}-u^{h}{\|}_{1}-\|u_{\infty }-u_{k_{h}}{\|}_{1}$$

which may be re-arranged to show the final result.  □

The error estimate presented in Lemma 1 enables us to interpret the error for any choice of $\left(S\text{,}k_{h}\right)$ and *k*, enabling the derivation of different permutations. A case of particular interest occurs when the projection $\pi^{h}$ is defined on a space *S* which satisfies the inf–sup condition, *i.e.* there is a β>0 such that [50,59,26,61],

(B.17) $$\underset{v^{h}\in X_{0}^{h}}{\sup}\frac{\int_{\Omega }\pi^{h}(g)\nabla \cdot v^{h}}{\|v^{h}{\|}_{1}}⩾\beta \|\pi^{h}(g)\|\text{,}\;\beta >0\text{,}\;g\in L^{2}(\Omega )\text{.}$$

In this case, the estimates in Lemma 1 may be simplified as shown in Corollary 1. Corollary 1 *Using the estimate in* Lemma 1*, and supposing the subspace S corresponding to the projection operator* $\pi^{h}:L^{2}(\Omega )\to S$ *is selected such that the inf–sup condition in Eq.* (B.17) *holds, then the weakly penalized form satisfies (for* $k=k_{h}$*),*

(B.18) $$\|u_{k_{h}}-u^{h}{\|}_{1}⩽C\underset{y^{h}\text{,}z^{h}\in X_{0}^{h}}{\inf}\left\{\|\pi^{h}(\nabla \cdot u_{k_{h}}^{⊥})-\nabla \cdot u_{k_{h}}^{⊥}\|+\|u_{k_{h}}^{*}-y^{h}{\|}_{1}+\left(\frac{1}{k}+1\right)\|u_{k_{h}}^{⊥}-z^{h}{\|}_{1}\right\}\text{,}$$

*with* C>0 *being positive constants independent of h.* Proof From [61], we observe that the weakly divergence free subspace $X_{0\text{,}\mathrm{div}}^{h}$ for a projection $\pi^{h}$ onto a space *S* which satisfies the inf–sup condition satisfies,

(B.19) $$\underset{y^{h}\in X_{0\text{,}\mathrm{div}}^{h}}{\inf}\|u_{k_{h}}^{*}-y^{h}{\|}_{1}⩽\left(\frac{\sqrt{d}}{\beta }+1\right)\underset{y^{h}\in X_{0}^{h}}{\inf}\|u_{k_{h}}^{*}-y^{h}{\|}_{1}\text{.}$$

As a consequence of this extension, we also observe that,

infyh∈X0h‖ukh-yh‖1=infyh∈X0,divhzh∈X0,divh⊥‖ukh∗-yh+ukh⊥-zh‖1⩾infzh∈(X0,divh)⊥‖ukh⊥-zh‖1-infyh∈X0,divh‖ukh∗-yh‖1

and hence

(B.20) $$\underset{z^{h}\in {\left(X_{0\text{,}\mathrm{div}}^{h}\right)}^{⊥}}{\inf}\|u_{k_{h}}^{⊥}-z^{h}{\|}_{1}⩽\underset{z^{h}\in X_{0}^{h}}{\inf}\|u_{k_{h}}^{⊥}-z^{h}{\|}_{1}+\left(\frac{\sqrt{d}}{\beta }+2\right)\underset{y^{h}\in X_{0}^{h}}{\inf}\|u_{k_{h}}^{*}-y^{h}{\|}_{1}$$

enabling the extension seen in the corollary.  □

From Corollary 1, it is clear that, when the inf–sup condition holds, optimal convergence rates may be obtained by applying standard interpolation theory for $X_{0}^{h}$ and the projection $\pi^{h}:L^{2}(\Omega )\to S$. While the PL, LM and weakly penalized forms may exploit this, the penalty formulation is subject to stricter conditions on $\pi^{h}$, namely $\pi^{h}:L^{2}(\Omega )\to L^{2}(\Omega )$. Consequently, the error bound for this projection may be expressed as seen in Corollary 2. Corollary 2 *Using the estimate in* Lemma 1*, and supposing the subspace* $S=L^{2}(\Omega )$ *corresponding to the projection operator* $\pi^{h}:L^{2}(\Omega )\to S$ *is selected, then the weakly penalized form satisfies (for* $k=k_{h}$*),*

(B.21) ‖ukh-uh‖1⩽Cinfyh∈X0,divhzh∈(X0,divh)⊥‖ukh∗-yh‖1+1k+1‖ukh⊥-zh‖1,

*where* $X_{0}^{h}=X_{0\text{,}\mathrm{div}}^{h}⊕{\left(X_{0\text{,}\mathrm{div}}^{h}\right)}^{⊥}$ *and* $X_{0\text{,}\mathrm{div}}^{h}≔\{v^{h}\in X_{0}^{h}|\;\|\nabla \cdot v^{h}\|=0\}$*.*

Here we note that $X_{0\text{,}\mathrm{div}}^{h}$ in Corollary 2 can be quite restrictive, yielding deterioration in the rate of convergence.

## Appendix C Derivation of the residual modifications

As mentioned before, the weakly penalized formulation is a generalization of the PL method, and as such should be able to present similar nonlinear behavior. To this end, the residual of the weakly penalized approach in Sections 2.5.3 and 2.5.4 is modified based on the residual derived from the PL method, in order to achieve the same nonlinear convergence. The static condensation used in the weakly penalized system follows the linear algebra used in this section in order to eliminate the pressure variable from the PL formulation. We can therefore use the PL formulation in order to study and understand the sources of poor nonlinear convergence for the weakly penalized formulation.

For later comparison, we first present the linearized system for the weakly penalized formulation, when the SNR scheme is applied, without the modifications introduced in this work. Based on Eq. (32), the update $\delta U^{n}$ for the $n^{\text{th}}$ iteration of the weakly penalized formulation is given by

(C.1) $$J(U^{\beta })\delta U^{n}=-R(U^{n})\text{,}$$

where the Jacobian is computed at a previous iteration β (the parameter $\alpha^{n}$ will be added in later through the minimization step). Using Eq. (34), the linearized system for the weakly penalized formulation can be written as

(C.2) $$(A^{\beta }+k{\left[B^{\beta }\right]}^{T}M^{-1}B^{\beta })\delta U^{n}=-R_{A}(U^{n})+k{\left[B^{n}\right]}^{T}M^{-1}R_{J}^{n}\text{.}$$

The linearized system for the ${\left(n+1\right)}^{\text{th}}$ iteration of the PL method when the SNR scheme (Section 2.5.4) is applied, can be expressed as

(C.3) $$\left(\begin{array}{cc} A^{\beta } & {\left[B^{\beta }\right]}^{T}\\ B^{\beta } & \frac{-1}{k}M \end{array}\right)\left(\begin{array}{c} \delta U^{n}\\ \delta p^{n} \end{array}\right)=\left(\begin{array}{c} -R_{A}^{n}-{\left[B^{n}\right]}^{T}p^{n}\\ -R_{J}^{n}+\frac{1}{k}Mp^{n} \end{array}\right)\text{,}$$

where we use the Jacobian matrix of the PL system (Eq. (A.8)) computed at a previous iteration β in the SNR iterative process. The updates for the displacement and pressure at the $n^{\text{th}}$ iteration are given by vectors $\delta U^{n}$ and $\delta p^{n}$. The right hand side of the matrix equation (C.3) denotes the residual at the $n^{\text{th}}$ iteration, where $R_{A}^{n}$ denotes the residual used in Eq. (35) and $R_{J}^{n}$ refers to the residual defined in Eq. (21).

The matrix Eq. (C.3) can be decomposed into the following equations:

(C.4) $$A^{\beta }\delta U^{n}+{\left[B^{\beta }\right]}^{T}\delta p^{n}=-R_{A}^{n}-{\left[B^{n}\right]}^{T}p^{n}\text{,}$$

(C.5) $$\delta p^{n}=kM^{-1}B^{\beta }\delta U^{n}+kM^{-1}R_{J}^{n}-p^{n}\text{.}$$

Combining the two equations we obtain

(C.6) $$(A^{\beta }+k{\left[B^{\beta }\right]}^{T}M^{-1}B^{\beta })\delta U^{n+1}=-R_{A}^{n}+{\left[B^{\beta }\right]}^{T}p^{n}-k{\left[B^{\beta }\right]}^{T}M^{-1}R_{J}^{n}-{\left[B^{n}\right]}^{T}p^{n}\text{.}$$

Note that if β=n always, this equation matches Eq. (C.2) of the weakly penalized approach thus the convergence behavior of the two formulations should be the same.

Taking into account Eq. (C.5), we can see that $p^{n}=p^{n-1}+\delta p^{n-1}=kM^{-1}(B^{\beta }\delta U^{n-1}+R_{J}^{n-1})$, which represents the linearized estimate for $R_{J}^{n}$. Using this estimate,

(C.7) $$(A^{\beta }+k{\left[B^{\beta }\right]}^{T}M^{-1}B^{\beta })\delta U^{n+1}=-R_{A}^{n}-k{\left[B^{n}\right]}^{T}M^{-1}(B^{\beta }\delta U^{n-1}+R_{J}^{n-1})+k{\left[B^{\beta }\right]}^{T}M^{-1}(R_{J}^{n-1}+B^{\beta }\delta U^{n-1}-R_{J}^{n})\text{.}$$

The last formulation can be used for a better understanding of the poor nonlinear convergence behavior of the weakly penalized formulation.

## Footnotes

1 In this paper, we consider only quadrilateral and hexahedral element types.

2 Default values used $\gamma =3/4\text{,}S_{1}=100$ for stiff problems, and *ITER*=15.

3 Note that, as the traction t typically depends on the physical domain, the operator $F_{\tau }$ often depends on u and is included in the Jacobian.

4 The original formulation proposed by Guccione et al. does not use the isochoric split of the deformation gradient [44]. Although this might incur some error, it is not expected to alter the trend of the results presented in this work, therefore the original version of the constitutive law was used, avoiding the required reparameterization of the isochoric version.

5 We note that in the examples presented in this work, the cost of computing the Jacobian is larger than the Newton–Raphson solution process, due to the quadratic interpolation used for the displacement and the higher order quadrature rule applied.

## References

[1] Hunter P., Pullan A., Smaill B. (2003). Modeling total heart function. Annu. Rev. Biomed. Eng..

[2] Mirsky I. (1973). Ventricular and arterial wall stresses based on large deformation analyses. Biophys. J..

[3] Guccione J., Costa K., McCulloch A. (1995). Finite element stress analysis of left ventricular mechanics in the beating dog heart. J. Biomech..

[4] Costa K., Holmes J., McCulloch A. (2001). Modelling cardiac mechanical properties in three dimensions. Philos. Trans. R. Soc. London A.

[5] Holzapfel G., Ogden R. (2009). Constitutive modelling of passive myocardium: a structurally based framework for material characterization. Philos. Trans. R. Soc. A.

[6] Hunter P., Smaill B. (1988). The analysis of cardiac function: a continuum approach. Prog. Biophys. Molec. Biol..

[7] LeGrice I., Smaill B., Chai L., Edgar S., Gavin J., Hunter P. (1995). Laminar structure of the heart: ventricular myocyte arrangement and connective tissue architecture in the dog. Am. J. Physiol. Heart Circ. Physiol..

[8] Sands G., Gerneke D., Hooks D., Green C., Smaill B., LeGrice I. (2005). Automated imaging of extended tissue volumes using confocal microscopy. Microsc. Res. Technol..

[9] Lamata P., Niederer S., Nordsletten D., Barber D., Roy I., Hose D.R., Smith N. (2011). An accurate, fast and robust method to generate patient-specific cubic Hermite meshes. Med. Image. Anal..

[10] Niederer S., Plank G., Chinchapatnam P., Ginks M., Lamata P., Rhode K., Rinaldi C., Razavi R., Smith N. (2011). Length-dependent tension in the failing heart and the efficacy of cardiac resynchronization therapy. Cardiovasc. Res..

[11] Nordsletten D., Niederer S., Nash M., Hunter P., Smith N. (2011). Coupling multi-physics models to cardiac mechanics. Prog. Biophys. Molec. Biol..

[12] Sermesant M., Moireau P., Camara O., Sainte-Marie J., Andriantsimiavona R., Cimrman R., Hill D., Chapelle D., Razavi R. (2006). Cardiac function estimation from MRI using a heart model and data assimilation: advances and difficulties. Med. Image. Anal..

[13] Moireau P., Chapelle D., Tallec P.L. (2008). Joint state and parameter estimation for distributed mechanical systems. Comput. Methods Appl. Mech. Eng..

[14] Usyk T., Mazhari R., McCulloch A. (2000). Effect of laminar orthotropic myofiber architecture on regional stress and strain in the canine left ventricle. J. Elasticity.

[15] Usyk T., McCulloch A., Holzapfel G., Ogden R. (2002). Computational methods for soft tissue biomechanics. Biomechanics of Soft Tissue in Cardiovascular Systems.

[16] Omens J., MacKenna D., McCulloch A. (1993). Measurement of strain and analysis of stress in resting rat left ventricular myocardium. J. Biomech..

[17] Nash M., Hunter P. (2000). Computational mechanics of the heart – from tissue structure to ventricular function. J. Elasticity.

[18] Stevens C., Remme E., LeGrice I., Hunter P. (2003). Ventricular mechanics in diastole: material parameter sensitivity. J. Biomech..

[19] Smith N., Stevens C., Hunter P. (2005). Computational modeling of ventricular mechanics and energetics. Appl. Mech. Rev..

[20] Rossi S., Ruiz-Baier R., Pavarino L., Quarteroni A. (2012). Orthotropic active strain models for the numerical simulation of cardiac biomechanics. Int. J. Numer. Methods Biomed. Eng..

[21] Yin F., Chan C., Judd R. (1996). Compressibility of perfused passive myocardium. Am. J. Physiol..

[22] Usyk T., LeGrice I., McCulloch A. (2002). Computational model of three-dimensional cardiac electromechanics. Comput. Visual Sci..

[23] Kerckhoffs R., Bovendeerd P., Kotte J., Prinzen K.S.F., Arts T. (2003). Homogeneity of cardiac contraction despite physiological asynchrony of depolarization: a model study. Ann. Biomed. Eng..

[24] Göktepe S., Acharya S., Wong J., Kuhl E. (2011). Computational modeling of passive myocardium. Int. J. Numer. Methods. Biomed. Eng..

[25] Vetter F., McCulloch A. (2000). Three-dimensional stress and strain in passive rabbit left ventricle: a model study. Ann. Biomed. Eng..

[26] Brezzi F., Fortin M. (1991). Mixed and Hybrid Finite Element Methods.

[27] Suri M. (1996). Analytical and computational assessment of locking in the hp finite element method. Comput. Methods Appl. Mech. Eng..

[28] Bathe K.-J. (1996). Finite Element Procedures.

[29] Holzapfel G. (2001). Nonlinear Solid Mechanics, A Continuum Approach for Engineering.

[30] Fried I. (1974). Finite element analysis of incompressible material by redidual energy balancing. Int. J. Solids Struct..

[31] Hughes T. (1980). Generalization of selective integration procedures to anisotropic and nonlinear media. Int. J. Numer. Methods Eng..

[32] Moran C.S.B., Ortiz M. (1990). Formulation of implicit finite element methods for multiplicative finite deformation plasticity. Int. J. Numer. Methods Eng..

[33] Simo J.C., Taylor R.L., Pister K.S. (1985). Variational and projection methods for the volume constraint in finite deformation elasto-plasticity. Comput. Methods Appl. Mech. Eng..

[34] Zienkiewicz J.T.O., Taylor R. (1971). Reduced integration technique in general analysis of plates and shells. Int. J. Numer. Methods Eng..

[35] Malkus T.H.D. (1978). Mixed finite element methods – reduced and selective integration techniques: a unification of concepts. Comput. Method Appl. M..

[36] Glowinski P.L.T.P. (1989). Augmented Lagrangian and operator-splitting methods in nonlinear mechanics.

[37] Simo J.C., Taylor R.L. (1991). Quasi-incompressible finite elasticity in principal stretches. continuum basis and numerical algorithms. Comput. Methods Appl. Mech. Eng..

[38] Herrmann L. (1965). Elasticity equations for incompressible and nearly incompressible materials by a variational theorem. AIAA J..

[39] Taylor L.H.R., Pister K. (1968). On a variational theorem for incompressible and nearly incompressible orthotropic elasticity. Int. J. Solids Struct..

[40] Key S.W. (1969). A variational principle for incompressible and nearly-incompressible anisotropic elasticity. Int. J. Solids Struct..

[41] Oden J.K.J. (1970). Numerical analysis of finite axisymmetric deformations of incompressible elastic solids of revolution. Int. J. Solids Struct..

[42] Shariff M.H.B.M. (1997). An extension of herrmanns principle to nonlinear elasticity. Appl. Math. Model..

[43] Nagtegaal J.C., Parks D.M., Rice J.R. (1974). On numerically accurate finite element solutions in the fully plastic range. Comput. Methods Appl. Mech. Eng..

[44] Thorvaldsen T., Osnes H., Sundnes J. (2005). A mixed finite element formulation for a non-linear, transversely isotropic material model for the cardiac tissue. Comput. Methods Biomech. Biomed. Eng..

[45] Bonet J., Wood R. (2008). Nonlinear Continuum Mechanics for Finite Element Analysis.

[46] Weiss J., Maker B., Govindjee S. (1996). Finite element implementation of incompressible transversely isotropic hyperelasticity. Comput. Methods Appl. Mech. Eng..

[47] Sussman T., Bathe K. (1987). A finite element formulation for nonlinear incompressible elastic and inelastic analysis. Comput. Struct..

[48] Chen J., Han W., Wu C., Duan W. (1997). On the perturbed Lagrangian formulation for nearly incompressible and incompressible hyperelasticity. Comput. Methods Appl. Mech. Eng..

[49] Chang T., Saleeb A., Li G. (1991). Large strain analysis of rubber-like materials based on a perturbed lagrangian variational principle. Comput. Mech..

[50] Bercovier M. (1978). Perturbation of mixed variational problems. Applications to mixed finite element methods. RAIRO.

[51] Liu C.H., Hofstetter G., Mang H.A. (1994). 3d finite element analysis of rubber-like materials at finite strain. Eng. Comput..

[52] Brink E.S.U. (1996). On some mixed finite element methods for incompressible and nearly incompressible finite elasticity. Comput. Mech..

[53] Miehe C. (1994). Aspects of the formulation and finite element implementation of large strain isotropic elasticity. Int. J. Numer. Methods Eng..

[54] Shamanskii V. (1967). A modification of Newton’s method. Ukrainian Math. J..

[55] McCormick M., Nordsletten D., Kay D., Smith N. (2012). Simulating left ventricular fluidsolid mechanics through the cardiac cycle under LVAD support. J. Comput. Phys..

[56] Zienkiewicz O., Taylor R., Zhu J. (2005). The Finite Element Method: Its Basis and Fundamentals.

[57] Simo J.C., Wriggers P., Taylor R.L. (1985). A perturbed lagrangian formulation for the finite element solution of contact problems. Comput. Methods Appl. Mech. Eng..

[58] Babuska I. (1973). The finite element method with Lagrangian multipliers. Numer. Math..

[59] Nicolaides R. (1982). Existence, uniqueness and approximation for generalized saddle point problems. SIAM J. Numer. Anal..

[60] Boland J., Nicolaides R. (1983). Stability of finite elements under divergence constraints. SIAM J. Numer. Anal..

[61] Quarteroni A., Valli A. (1994). Numerical Approximation of Partial Differential Equations.

[62] Crouzeix M., Raviart P. (1973). Conforming and nonconforming finite element methods for solving the stationary stokes equations I. RAIRO.

[63] Taber L., Yang M., Podszus W. (1996). Mechanics of ventricular torsion. J. Biomech..

[64] Korakianitis T., Shi Y. (2006). Numerical simulation of cardiovascular dynamics with healthy and diseased heart valves. J. Biomech..

[65] M. McCormick, Ventricular function under LVAD support (Dissertation), University of Oxford, 2011.

[66] D. Nordsletten, Fluid–solid coupling for the simulation of left ventricular mechanics (Dissertation), University of Oxford, 2009.

[67] Nordsletten D., McCormick M., Kilner P., Hunter P., Kay D., Smith N. (2011). Fluid–solid coupling for the investigation of diastolic and systolic human left ventricular function. Int. J. Numer. Methods Biomed. Eng..

[68] Nordsletten D., Kay D., Smith N. (2010). A non-conforming monolithic finite element method for problems of coupled mechanics. J. Comput. Phys..

[69] Richie G.R., Bullivant D., Blackett S., Hunter P. (2002). Modelling and visualising the heart. Comput. Visual Sci..

[70] Benzi M., Golub G., Liesen J. (2005). Numerical Solution of Saddle Point Problems. Acta Numer..

[71] Duvaut G., Lions J.L. (1972). Les inéquations en mécanique et en physique.

